# Technological functionality and system architecture of mobile health interventions for diabetes management: a systematic review and meta-analysis of randomized controlled trials

**DOI:** 10.3389/fpubh.2025.1549568

**Published:** 2025-02-20

**Authors:** Xinran Yu, Yifeng Wang, Zhengyang Liu, Euitay Jung

**Affiliations:** ^1^Graduate School, Major of Visual Design, Hanyang University, Seoul, Republic of Korea; ^2^School of Art, Shandong University of Finance and Economics, Jinan, Shandong, China

**Keywords:** mobile applications, diabetes management, system architecture, random controlled trials, meta-analysis

## Abstract

**Introduction:**

Despite advancements in digital health, systematic evaluations of mobile applications (Apps) for diabetes management are limited.

**Methods:**

Researchers conducted searches on PUBMED, EMBASE, COCHRANE, SCOPUS, and WEB OF SCIENCE from inception to August 2024. The researchers included randomized controlled trials (RCTs) that investigated the effectiveness of app-based interventions in health management among diabetic patients. Reviewers were paired and independently conducted the screening of studies, data extraction, and evaluation of study quality. The primary outcome of interest was the modification of hemoglobin A1c (HbA1c). The researchers utilized a random effects model to calculate the weighted mean differences (WMDs) and 95% confidence intervals (CIs) and used the I^2^ statistic to assess study heterogeneity. Publication bias for the primary outcomes underwent assessment. Studies were Appraised for quality using the Cochrane Risk of Bias assessment.

**Results:**

41 studies of 3911 initially identified articles that met the selection criteria. The results showed that Apps’ intervention significantly improved glycemic control in diabetic patients, with a mean reduction in HbA1c levels of 0.49% (95%CI: –0.65 to –0.32%) compared to standard care. The analysis also revealed that Apps enhanced patient self-management behaviors. Subgroup analyses failed to resolve heterogeneity, but studies consistently observed improved HbA1c levels. The quality assessment results indicated that most studies performed well in the completeness of outcome data and selective reporting.

**Discussion:**

This meta-analysis confirms that mobile health applications with practical technological functionalities and system architectures are beneficial in managing diabetes. These applications significantly reduced HbA1c levels and improved self-management behaviors. Although some studies exhibited a moderate risk of bias, the overall evidence supports using these applications as valuable tools in diabetes care. Future research should standardize application features, refine system architectures, and address bias issues to enhance.

**Systematic Review Registration:**

PROSPERO (CRD42023441365).

## 1 Introduction

Diabetes mellitus (DM) affects a staggering number of individuals worldwide, making it one of the most prevalent non-communicable diseases (1, 2). In 2023, researchers estimated that over 500 million people are living with diabetes, and this number continues to rise each year (3). Chronic hyperglycemia, which results from defects in insulin secretion, insulin action, or both, characterizes diabetes and often leads to severe complications (4). Effective diabetes management is crucial to mitigate its complications, which include cardiovascular diseases, neuropathy, nephropathy, and retinopathy (5). Diabetes management is complex and requires a multifaceted approach involving lifestyle modifications, regular monitoring of blood glucose levels, medication adherence, and patient education (6). Technological advancements, particularly the emergence of mobile health applications (Apps), have significantly influenced the landscape of diabetes care in recent years (7, 8). By improving hypertension and dyslipidemia, reducing blood glucose levels can significantly lower the risk of adverse clinical outcomes in patients with type 2 diabetes and effectively control treatment costs. Current clinical guidelines recommend that adult patients with type 2 diabetes aim to maintain glycated hemoglobin (HbA1c) levels below 7.0% (53 mmol/mol) as one of the key goals in glycemic management (9).

In recent years, several systematic reviews have explored the potential of digital health technologies in diabetes management. Developing specialized applications has provided diabetes patients with convenient and easily accessible tools. The use of mobile phones has become ubiquitous, and the development of specialized Apps offers convenient and accessible tools for individuals with diabetes (10). These Apps can perform various functions, including real-time monitoring of blood glucose levels, dietary tracking, physical activity monitoring, and providing personalized educational content (11). They also enable patients to share their health data with their healthcare providers, facilitating remote and timely medical advice (12). Studies have shown that well-designed diabetes management Apps can positively impact patients’ self-care behaviors and glycemic control (12, 13). For instance, researchers have found that apps incorporating reminder systems for medication adherence and goal-setting features improve treatment compliance (14). Moreover, some Apps use gamification elements to enhance user engagement and motivation, making the management process more enjoyable and sustainable (15, 16). However, despite the promising potential, there are several challenges to the widespread adoption and effective use of diabetes Apps (17, 18). Issues such as data accuracy, interoperability between different Apps and healthcare systems, and the digital divide among patients with varying levels of technological literacy require resolution (11).

Additionally, these Apps’ long-term efficacy and cost-effectiveness in clinical practice require further investigation (19). Recent reviews have emphasized the potential of digital health technologies to improve medication adherence and patient engagement while addressing the challenges of long-term sustainability and accessibility (20, 21). Georgieva et al. highlighted the need for personalized technological support and the importance of overcoming barriers to adoption among older adult populations (20). In contrast, another review focused on the role of digital tools in T2DM management, noting their ability to reduce logistical barriers like travel costs and enhance adherence through convenient follow-ups (21). These issues underscore the need for further research into digital health interventions’ functionality and system architecture (17, 18). Moreover, the study pointed out significant research gaps in existing technologies regarding their applicability across diverse cultural contexts, patient populations, and long-term outcomes. Although the studies provide valuable insights, their limitations form the basis for the present research. Specifically, systematic reviews do not comprehensively assess digital health interventions’ functionality, intervention models, and effectiveness in reducing glycated hemoglobin (HbA1c). Additionally, key issues such as user adoption rates, implementation feasibility, and the scalability of these technologies in various healthcare settings remain insufficiently explored. Furthermore, these interventions’ long-term clinical efficacy and cost-effectiveness in clinical practice require further investigation (19). Therefore, this study aims to address these research gaps by comprehensively analyzing mobile health intervention technologies for diabetes management, focusing on application functionality, system architecture, and effectiveness.

Unlike previous systematic reviews, this systematic review and meta-analysis focused on exploring how mobile health interventions’ technological functionalities and system architectures influence outcomes in diabetes management. Recent findings have highlighted the role of digital technologies, such as mobile apps and teleconsultations, in enhancing patient adherence and optimizing diabetes care while stressing the need for standardized protocols to guide their effective integration into healthcare systems (21). By systematically analyzing functionalities and comparing intervention models, this study provides practical insights for developing more efficient digital health technologies and integrating them into existing healthcare systems. This systematic review and meta-analysis comprehensively evaluate technological functionalities and system architectures of mobile health interventions for managing type 2 diabetes. Specifically, it examines the effectiveness of various intervention models in reducing glycated hemoglobin (HbA1c) levels and systematically analyzes diabetes management applications in terms of app functionality, intervention models, and technical implementation. Additionally, the study examines user adoption rates, implementation feasibility, and intervention coverage. By synthesizing evidence on the role of mobile applications in diabetes care, this research seeks to provide practical insights for optimizing digital health technologies and integrating them into routine clinical practice. Ultimately, it aims to improve health outcomes for adult patients with type 2 diabetes by enhancing the user experience and accessibility of mobile health interventions.

## 2 Methods

### 2.1 Protocol

This systematic review and meta-analysis were conducted strictly by the Preferred Reporting Items for Systematic Reviews and Meta-Analyses (PRISMA) guidelines (22). The review protocol was registered on PROSPERO and is publicly available (CRD42023441365).

### 2.2 Search strategy

A comprehensive electronic search was conducted in PubMed, Embase, Scopus, Web of Science, and the Cochrane Library using broad and MeSH search terms. The search period spanned from the inception of each database to November 2024. The optimized search strategy incorporated the following keywords: “type 2 diabetes,” “T2D,” “T2DM,” “diabetes,” “hyperglycemia,” and “hypertension.” Supplementary Table S1 reports the detailed search strategies.

### 2.3 Study selection

The researchers applied the following eligibility criteria for the review:

- Patients with type 1 or type 2 diabetes, regardless of gender, race, or nationality.
- The intervention involved patients who underwent mobile app-based interventions.
- The comparison included standard care without mobile app-based interventions.
- The primary outcome was the change in Hemoglobin A1c concentration, a percentage measure widely used for diagnosing and managing diabetes.
- The study utilized randomized controlled trials, providing the highest evidence for intervention efficacy.

If a specific study did not fulfill any inclusion criteria, the researchers excluded it from the analysis. The researchers also excluded case reports, technical notes, and letters to the editor. Two authors (X.Y. and Z.L.) independently screened the titles and abstracts of all identified citations, with a full-text review when the abstract was insufficient to determine whether the study met the inclusion or exclusion criteria. Y.W. reviewed the selected literature. The researchers resolved discrepancies by mutual consensus.

### 2.4 Data collection

From each included article, the researchers adopted a systematic and rigorous data extraction process to ensure the accuracy and reliability of the data. The extracted information encompassed the following:

- Publication details (author, year, country, study design, and sample size).
- Characteristics of the participants (age, sex, diabetes type, and diabetes duration).
- The details of the intervention (type, follow-up duration).
- Outcomes (HbA1c at baseline and follow-up).

Two researchers (X.Y. and Z.L.) independently performed data extraction, with Y.W. reviewing the results and resolving discrepancies through consultation and discussion (23).

#### 2.4.1 Data collection

The researchers appraised the risk of bias within the selected studies using the Cochrane Collaboration tool (24). This tool evaluates several key domains: the generation of random sequences, the concealment of allocation, the blinding of both participants and personnel, the blinding of outcome assessment, the handling of incomplete outcome data, the potential for selective reporting, and the presence of other biases. Two investigators (X.Y. and Z.L.) independently assigned each domain a low, unclear, or high-risk rating. Y.W. and the aforementioned investigators addressed disagreements in risk assessment through consensus discussions.

#### 2.4.2 Meta-analysis

The principal endpoint under investigation was the variation in glycated hemoglobin (HbA1c) level. A random-effects meta-analysis determined the mean difference (MD) in HbA1c alterations between the experimental and control cohorts. The researchers utilized the software Review Manager v5.4.1 to input the mean differences and corresponding standard deviations (SD) of HbA1c changes within each group, both for the experimental and control arms. In cases where studies reported only the differences between groups rather than within groups, the researchers solicited additional data from the original authors. When studies provided the standard error (SE) instead of the SD, the researchers applied a conversion formula to derive the SD. For those studies that offered 95% confidence intervals (CI) rather than SD, the SD was estimated based on the guidelines outlined in Chapter 7.7.3.2 of the Cochrane Handbook. Studies were omitted from the meta-analysis if the SD, SE, or 95% CI data were inaccessible and the authors failed to provide the necessary information upon request.

Using a random-effects model, the researchers aggregated the effect sizes and their standard deviations (SDs). Although this model can lead to broader confidence intervals for the point estimates, the researchers selected it to account for the expected variability due to disparities in participant demographics and research methods across studies. The researchers quantified the degree of heterogeneity among the studies using the I^2^ statistic, classifying it as follows: 0–40% signifies low heterogeneity, 30–60% suggests moderate heterogeneity, 50–90% denotes substantial heterogeneity, and 75–100% reflects considerable heterogeneity (25). The researchers assessed the stability of the findings through sensitivity analyses, which involved the iterative removal of individual studies, subsequent reevaluation of the dataset, and comparison of the outcomes with those from fixed-effects models. Furthermore, Cochran’s Q test was applied to evaluate the consistency of effect sizes, while publication bias was investigated by examining funnel plots and conducting Egger’s regression analysis.

The researchers categorized interventions as ineffective if they observed no statistically or clinically significant differences between groups. Secondary outcomes included blood pressure, lipid profiles (total cholesterol, HDL cholesterol, LDL cholesterol, and triglycerides), fasting glucose levels, medication adjustments, and anthropometric measures (e.g., BMI, weight, and waist circumference). The researchers conducted subgroup analyses to explore the impact of age, diabetes type, sample size, publication year, region, and intervention duration on the results. The researchers assessed publication bias using funnel plots and Egger’s linear regression test, considering *p*-values <0.05 as statistically significant.

## 3 Results

### 3.1 Study description

**Figure 1:**
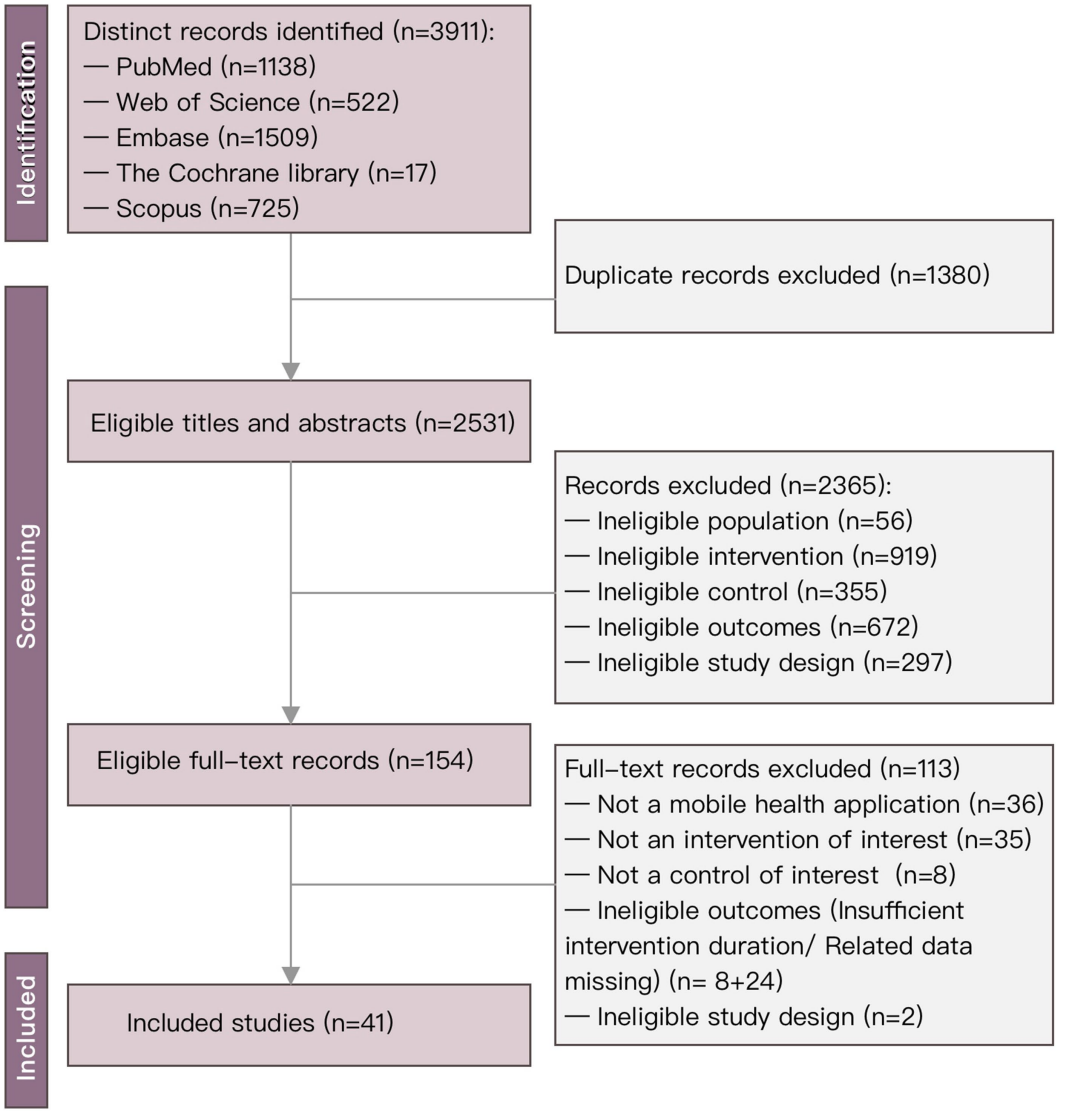
PRISMA flowchart of study selection.

Our initial literature search yielded 3,911 citations. After removing duplicates, we retained 604 unique records. Upon reviewing the titles and abstracts, we deemed 1,380 articles ineligible and excluded these publications. During the full-text screening, we excluded an additional 113 studies. Following the full-text assessment, we excluded another 113 studies, with the details of these exclusions in Supplementary Table S2. Finally, we selected 41 studies for eligibility. Ultimately, 41 studies met the inclusion criteria, as illustrated in the PRISMA flowchart in Figure 1. Details on compliance with PRISMA 2020 are summarized in the PRISMA checklist, shown in Supplementary Table S3. The types of diabetes covered include type 1 diabetes (T1D) and type 2 diabetes (T2D); the numbers of related studies are 11 and 23, respectively. In seven studies, the participants had both T1D and T2D.

The characteristics of the included studies are presented in Tables 1, 2. The included researches were mainly published from 2011 to 2023, among which the number was the largest from 2019 to 2020 (12 studies, 29.27%). Most trials were conducted in China (*n* = 7) and the United States (*n* = 6). Despite the uneven distribution of studies, this systematic review included research from all six continents. Asia contributed 16 studies, Europe 14, North America 9 studies, Africa 1 study, and Oceania 1. Based on World Bank criteria, economic status revealed that 25 studies were from high-income countries, 10 from upper-middle-income countries, and 6 from lower-middle-income countries (26).

**Table 1:** Characteristics of included studies that incorporated smartphone App interventions.

Application type	References	Country	Participants (I/C)	Age years (I/C)	I	C	Diabetes type	Diabates duration years (I/C)	Outcomes^a^
Medication Adherence Apps (MAA)	Kamat et al. (27)	India	59/59	47.2 ± 5.8/47.6 ± 6.6	30/29	29/30	T2D	4.2 ± 2.3/4.0 ± 2.2	①②③④
	Huang et al. (31)	Singapore	25/25	51.5/52	9/13	11/8	T2D	NR	①⑧⑦⑤③
Telemedicine Apps (TA)	Bujnowska-Fedak et al. (48)	Poland	50/50	53.1 ± 25.2/57.5 ± 27.4	26/21	25/23	T2D	8.1 ± 7.6/7.7 ± 6.8	①③④⑤⑥
	Klee et al. (47)	Switzerland	28/27	13.6 ± 2.4/13.7 ± 2.4	21/7	10/17	T1D	7.5 ± 4.0/5.5 ± 3.25	①
	Crowley et al. (54)	US	25/25	60 ± 8.4/60 ± 9.2	25/0	23/2	T2D	12 (IQR = 13)/12 (IQR = 9)	①④⑩
	Baron et al. (58)	UK	45/36	58.2 ± 13.6/55.8 ± 13.8	31/14	14/22	T1D/T2D	NR	①④⑪
	Ruiz De Adana et al. (59)	Spain	163/167	33.78 ± 9.77/36.22 ± 10.78	90/73	94/73	T1D	≥2	①
Behavioral Support Apps (BSA)	Goyal et al. (32)	Canada	46/46	14.1 ± 1.7/13.9 ± 1.5	21/25	20/26	T1D	7.1 ± 3.2/6.6 ± 3.2	①
	Knox et al. (55)	UK	24/25	10.40 ± 1.1/10.89 ± 0.9	10/14	17/8	T1D	≥0.25	①
	Sevick et al. (63)	US	131/132	NR	38/93	46/86	T2D	NR	①⑧⑦⑨④②③
	Ruissen et al. (28)	Spain	111/115	51.5 ± 13.2/51.5 ± 10.9	71/40	73/42	T1D/T2D	16.9 ± 11.6/17.9 ± 12.3	①
	Hilmarsdóttir et al. (61)	Iceland	15/15	NR	9/6	10/5	T2D	4.9 ± 5.1/7.4 ± 4.4	①②③④⑨⑦⑧⑫
Education and Knowledge Support Apps (EKSA)	Buysse et al. (49)	Belgium	81/72	37 ± 14.7/38 ± 13.2	43/38	34/38	T1D/T2D	NR	①
	Zhou et al. (62)	China	50/50	55.0 ± 13.1/53.5 ± 12.4	27/23	30/20	T1D/T2D	6.65 ± 5.14/6.63 ± 5.06	①③④⑧
	Or et al. (56)	Hong Kong	33/30	69.3 ± 9.7/69.7 ± 10.2	14/19	6/24	T2D	13.9 ± 18.3/10.7 ± 8.1	①④
	Moattari et al. (64)	Iran	22/22	23.35	NR	NR	T1D	NR	①⑨⑦⑧⑫
Comprehensive Management Apps (CMA)	Rossi et al. (53)	Italy	64/64	38.4 ± 10.3/34.3 ± 10.0	29/35	31/33	T1D	16.2 ± 10.0/15.0 ± 8.4	①②④⑤⑦⑧⑨
	Gunawardena et al. (36)	Sri Lanka	35/32	52 ± 11.7	22/13	18/14	T2D	11 ± 6/11 ± 7	①
	Lee et al. (45)	Korea	91/92	56.70 ± 7.16/55.55 ± 8.56	61/30	62/30	T2D	9.74 ± 6.01/9.72 ± 7.09	①
	Zhai et al. (33)	China	60/58	54.12 ± 11.1/55.64 ± 14.2	30/30	28/30	T2D	11.7 ± 5.49/12.1 ± 3.25	①
	Sun et al. (72)	China	44/47	67.9 ± 3.71/68.04 ± 4.45	19/25	18/29	T2D	NR	①④⑤
	Tang et al. (35)	US	202/213	54.0 ± 10.7/53.5 ± 10.2	119/83	130/83	T2D	NR	①④⑦
	Kumar et al.(46)	India	150/150	18–65 years	90/60	90/60	T2D	> 1	①
	Wang et al. (43)	China	106/106	52.6 ± 9.1/54.7 ± 10.3	64/46	52/58	T2D	8.1 ± 7.6/7.7 ± 6.8	①③⑨⑤⑦⑧④
	Han et al. (34)	China	212/206	52.1 ± 9.2/51.8 ± 8.3	62/150	48/158	T2D	7.4 ± 1.5/7.2 ± 1.5	①③④⑨⑤⑦⑧
	Quinn et al. (50)	US	62/56	52 ± 8.0/53.2 ± 8.4	31/31	28/28	T2D	8.2 ± 5.3/9.0 ± 7.0	①③④⑧⑦⑨⑤
	Zamanillo-Campos et al. (66)	Spain	96/111	63 ± 10/61 ± 12	61/35	74/37	T2D	8.7 ± 5.1/8.2 ± 5.1	①
	Waki et al. (65)	Japan	15/7	55.9 ± 10.8/59.7 ± 9.9	13/2	4/3	T2D	>5	①③④⑧⑦⑨
	Zhang et al. (44)	China	78/78	55 ± 11/52 ± 10	50/28	49/29	T1D/T2D	11.2 ± 5.6/12.7 ± 7.1	①
	Riangkam et al. (37)	Thailand	43/43	50.30 ± 1.11/52.65 ± 0.88	25/18	27/16	T2D	7.1 ± 5.64/7.1 ± 5.44	①
	Wang et al. (70)	China	60/60	45.13 ± 7.83/45.8 ± 8.38	33/37	31/29	T2D	NR	①
	Kirwan et al. (38)	Australia	36/36	35.97 ± 10.67/34.42 ± 10.26	19/17	9/27	T1D	19.69 ± 9.64/18.19 ± 9.77	①
	Iljaz et al. (67)	Slovenia	58/60	56.3 ± 10.5/54.7 ± 11.1	36/22	37/23	T2D	5.1 ± 5.7/5.7 ± 4.8	①④⑨⑧⑦③⑫
	Jeffrey et al. (68)	Canada	22/22	13.98 ± 1.57/13.98 ± 1.76	11/11	16/6	T1D	6.08 ± 4.14/6.44 ± 4.45	①
	Anzaldo-Campos et al. (51)	Mexico	102/100	51.5 ± 11.4/52.5 ± 9.7	39/63	38/62	T2D	NR	①⑤⑨⑧⑦③④
	Bisio et al. (60)	US	57/23	33.44/39.91	23/34	12/11.	T1D	15.85/15.26	①
	Charpentier et al. (57)	France	59/61	31.6 ± 12.5/36.8 ± 14.1	22/37	21/40	T1D	14.7 ± 9.1/16.9 ± 10.5	①
	Chatzakis et al. (69)	Greece	40/40	13.5 ± 2.8/13.5 ± 2.8	NR	NR	T1D	NR	①
	Derkaoui et al. (71)	Morocco	32/30	14 ± 6/17 ± 6	19/13	14/16	T1D	4 ± 4/6 ± 4	①
	Forjuoh et al. (73)	US	99/95	57.7 ± 10.3/58.5 ± 11.9	46/53	42/53	T2D	NR	①
	Franc et al. (52)	France	231/221	39.1/38.3	NR	NR	T1D/T2D	17.8/1.8	①

a ① Hemoglobin A1c, ② weight, ③ body mass index, ④ blood pressure (systolic/diastolic), ⑤ total cholesterol (mg/dL), ⑥ creatinine (mg/dL), ⑦ HDL-cholesterol (mg/dL), ⑧ LDL-cholesterol (mg/dL), ⑨ triglycerides (mg/dL), ⑩ Self-Care Inventory–Revised, ⑪ daily insulin dose, ⑫ cholesterol.

**Table 2:** Summary of HbA1c data by intervention.

Application type	References	Intervention	Follow-up (month)	HbA1c at baseline (I)	HbA1c at follow-up (I)	HbA1c at baseline (C)	HbA1c at follow-up (C)
MAA	Kamat et al. (27)	The “incentive group” used the mobile app which provided up to four medication reminders per dose, pill identification and counting, and financial rewards based on adherence scores; reminders.	6	9.0 ± 0.3	7.3 ± 0.2	9.0 ± 0.3	8.2 ± 0.3
	Huang et al. (31)	Medisafe app for medication management; medication scheduling, reminders, tracking, data sharing, adherence assessments; research team as “Medfriend” on the app.	3	8.7 ± 2.4	9.0 ± 1.6	8.6 ± 1.5	9.4 ± 2.4
TA	Bujnowska-Fedak et al. (48)	Weekly data transmission; includes blood glucose levels, insulin doses, event specifics; real-time alerts; trend data presentation; trend data presentation.	24	7.63 ± 1.53	7.37 ± 1.27	7.61 ± 1.65	7.43 ± 1.49
	Klee et al. (47)	Monthly blood glucose reviews and feedback for treatment adaptation; remote access to blood glucose data; bolus calculator for insulin dosing; meal tracking; data management and historical overview.	3	8.1 ± 1.4	7.8 ± 1.0	8.1 ± 0.9	8.3 ± 0.8
	Crowley et al. (54)	Telemonitoring; Self-management support covering SMBG, hypoglycemia, medication use, diet, exercise, and complications; option to repeat modules; physician-guided management	6	10.5 ± 0.2	9.2 ± 0.4	10.5 ± 0.2	10.2 ± 0.4
	Baron et al. (58)	self-monitoring, mobile-phone data transmissions, graphical and nurse-initiated feedback, and educational calls.	9	9.07 ± 1.72	8.56 ± 1.64	8.88 ± 1.68	8.93 ± 1.61
	Ruiz De Adana et al. (59)	Includes insulin doses, carbohydrates consumed, physical activity, and other health data inputs; generates charts, graphs, and statistics for patient and clinician use.	6	7.0 ± 0.8	7.0 ± 0.8	7.0 ± 0.7	7.0 ± 0.7
BSA	Goyal et al. (32)	Wireless blood glucose reading transfer, out-of-range blood glucose trend alerts, coaching around out-of-range trend causes and fixes, point-based incentive system.	12	8.96 ± 0.7	8.96 ± 1.3	8.92 ± 0.6	8.96 ± 1.2
	Knox et al. (55)	STAK-D website; physical activity goal setting; feedback; knowledge enhancement; self-efficacy enhancement for diabetes self-management; interactions with project researchers.	6	7.15 ± 0.85	7.33 ± 1.38	7.00 ± 1.18	7.24 ± 0.77
	Sevick et al. (63)	Mobile device (PDA) with dietary self-monitoring app; Dietary program adjusts targets based on metabolic rate; customizable meal entries; Real-time feedback on diet and physical activity via PDA.	6	7.7 ± 2.2	7.1 ± 1.3	7.5 ± 1.7	7.3 ± 1.6
	Ruissen et al. (28)	Goal setting, tracking goal progress, automatic reminders, barrier identification, providing psychoeducation and targeted interventions; reminders are automatically triggered based on the set goals and tracked progress.	10.05	7.7 ± 1.3	7.3 ± 1.1	7.8 ± 1.3	7.7 ± 1.1
	Hilmarsdóttir (61)	Digital lifestyle program; goal setting, self-monitoring, health-related tasks; nutrition; physical activity; stress management; clinic; gamified technology with health points and rewards, standard guidance and support; individualized encouragement based on app activity.	6	7.7 ± 2.0	7.0 ± 1.4	7.8 ± 1.9	7.7 ± 1.4
EKSA	Buysse et al. (49)	Immediate access to tele-education platform; education communication; user management via eConnecta platform allowing specific access to patient data; viewing data through tables and graphs; receive educational feedback via platform.	24	8.3 ± 3.7	7.3 ± 3.2	7.9 ± 3.3	7.4 ± 3.3
	Zhou et al. (62)	Educational content; Regular advice and feedback; personalized feedback based on individual data entries; alerts for critical thresholds.	3	9.86 ± 2.38	7.91 ± 1.58	9.76 ± 2.51	8.97 ± 2.08
	Or et al. (56)	Continuous monitoring and recording; educational materials available on demand; visual feedback; structured data presentation in graphs and tables.	3	7.4 ± 0.6	7.2 ± 0.8	7.3 ± 0.7	7.0 ± 0.7
	Moattari et al. (64)	The site includes a variety of educational materials; interactivity; communication with healthcare providers; monitoring tools.	3	9.10 ± 1.29	7.07 ± 1.19	9.42 ± 1.78	8.82 ± 1.31
CMA	Rossi et al. (53)	The DID system is a mobile software used as a carbohydrate/insulin bolus calculator that supports patients in managing carbohydrate counting through a food atlas, recording SMBG measurements, and calculates the most appropriate insulin dose to be injected at each meal.	6	8.4 ± 0.1	7.9 ± 0.1	8.5 ± 0.1	8.1 ± 0.1
	Gunawardena et al. (36)	Smart Glucose Manager; reminds patients to monitor blood glucose, take medication, eat, and exercise as scheduled; real-time glucose values; provides options to view and export data; data calculation.	6	9.52 ± 1.10	7.2 ± 0.76	9.44 ± 1.37	8.17 ± 0.85
	Lee et al. (45)	Use of a digital integrated health care platform without assistance.	12	7.47 ± 0.38	7.2 ± 0.64	7.45 ± 0.36	7.52 ± 0.8
	Zhai et al. (33)	Sync read of glucose values via cable-connected glucometer; support for diabetes self-management including diet advice, emotional management, medication guidance; online instruction.	6	8.86 ± 1.15	6.7 ± 1.06	9.05 ± 1.23	7.22 ± 1.02
	Sun et al. (72)	Bi-weekly medical advice and glucose monitoring reminders; monthly dietary recommendations based on dietary intake data from app; exercise guidance based on texted pedometer data.	6	7.84 ± 0.73	6.84 ± 0.77	7.88 ± 0.64	7.77 ± 0.87
	Tang et al. (35)	Wireless remote monitoring tools; online logging of diet, activity, blood pressure, insulin doses, weight; secure messaging; medication adjustments; personalized education via PHR.	12	9.24 ± 1.59	8.10 ± 1.68	9.28 ± 1.74	8.33 ± 1.81
	Kumar et al. (46)	Alerts; medication reminders; caloric tracking, health reports, educational content, real-time tracking; easy navigation and personalized settings.	6	7.36 ± 1.04	7.10 ± 0.96	7.84 ± 1.37	7.97 ± 1.37
	Wang et al. (43)	Web-based telemedicine system (U-Healthcare website); Bi-weekly analysis and advice on blood glucose, diet, exercise; advice personalized.	6	7.9 ± 0.7	6.8 ± 0.7	8.0 ± 0.8	7.4 ± 1.3
	Han et al. (34)	Real-time data upload, color-coded display of blood glucose levels, feedback on behavioral impacts, and secure communication with healthcare providers.	6	7.95 ± 0.9	7.38 ± 1.1	8.03 ± 0.9	7.98 ± 0.1
	Quinn et al. (50)	Real-time automated educational, behavioral, and motivational messaging based on patient-entered data; personalized feedback; health records.	12	9.9 ± 2.1	7.9 ± 1.7	9.2 ± 1.7	8.5 ± 1.8
	Zamanillo-Campos et al. (66)	DiabeText system sends automated text messages; including medication adherence, diet, and physical activity; integrates patient-generated data and routinely collected clinical data.	3	9.1 ± 1.3	7.7 ± 1.3	9.0 ± 1.0	7.6 ± 1.2
	Waki et al. (65)	Automated data system feedback; text and voice input, automated advice; food recording and dietary evaluation (FoodLog): input by photo or text, nutritional evaluation, and diet advice.	3	7.3 ± 1.0	6.7 ± 0.7	6.4 ± 0.5	6.7 ± 0.5
	Zhang et al. (44)	App-based learning, diet, exercise, medication, insulin use; contact clinicians online.	6	9.14 ± 1.13	8.04 ± 1.38	9.14 ± 1.13	7.80 ± 1.14
	Riangkam et al. (37)	Deliver diabetes-related knowledge; text content; quizzes; video links; self-monitoring of blood glucose; medication reminders; emergency call.	3	7.8 ± 0.50	7.2 ± 0.45	7.9 ± 0.53	7.7 ± 0.61
	Wang et al. (70)	Hand-held clinic; blood glucose monitoring; dietary recording; exercise guidance; personal health reports; professional knowledge; community; health guide; health advice; follow-up.	6	8.62 ± 2.33	7.12 ± 2.01	8.68 ± 2.26	7.92 ± 2.15
	Kirwan et al. (38)	Log blood glucose levels, insulin dosages, medications, diet, and physical activities; View data on customizable graphs and export via email; Weekly review of logged data by a CDE and Personalized text-message communication once a week for the first 6 months.	9	9.08 ± 1.18	7.80 ± 0.75	8.47 ± 0.86	8.58 ± 1.16
	Iljaz et al. (67)	Bi-weekly data recording; Every 6–8 weeks COOP-WONCA charts completion; Individualized care interface; Educational material tailored to patient needs; Automated reminders and emergency warnings via email and SMS.	10	7.1 ± 1.5	6.4 ± 0.9	6.8 ± 1.2	6.7 ± 1.5
	Jeffrey et al. (68)	Educational and practical content; Image recognition algorithm; co-designed with diabetes educators and dietitians; Android and iOS compatibility; iterative refinement based on user feedback.	3	8.41 ± 1.84	8.06 ± 1.43	8.35 ± 1.32	8.80 ± 1.60
	Anzaldo-Campos et al. (51)	Glucose level checks; data uploaded to diabetes registry system; interactive surveys, text messages, educational videos, brochures on cell phone; automated reminders for surveys, alerts for abnormal glucose levels or missed appointments; access to culturally appropriate diabetes care videos.	10	11.19 ± 2.03	8.17 ± 2.13	10.90 ± 2.01	9.60 ± 2.71
	Bisio et al. (60)	CGM connectivity, smart bolus calculator, hypoglycemia and exercise risk warnings, and biweekly treatment optimization; cloud-based infrastructure for updates and monitoring; communication between smart insulin pens and the app to record insulin administration.	3.5	7.41 ± 1.18	7.11 ± 1.61	7.4 ± 1.8	7.1% ± 0.9
	Charpentier et al. (57)	Diabeo software features a bolus calculator with validated algorithms, incorporating SMPG levels, carbohydrate counts, physical activity, and personalized insulin dose adjustments for prandial and basal insulin. It also suggests adjustments for carbohydrate ratio, long-acting insulin, or pump basal rates based on SMPG targets.	6	9.11 ± 1.14	8.41 ± 1.04	8.91 ± 0.90	9.10 ± 1.16
	Chatzakis et al. (69)	Daily glucose measurements; database of 7,000 foods with carbohydrate and lipid content; adjustable parameters (insulin correction factor, carbohydrate factor, lipid factor); personalized bolus dose calculations based on meal content, physical activity, and individual insulin requirements.	12	7.9 ± 0.5	7.0 ± 0.4	7.8 ± 0.5	7.5 ± 0.5
	Derkaoui et al. (71)	Daily; educational on hypoglycemia and hyperglycemia management, insulin injections, dosing adaptation, blood glucose level entry, insulin dosage recording, physical activity, and diet logging.	3	8.3 ± 2.4	7.4 ± 1.5	8.2 ± 2.0	8.0 ± 1.8
	Forjuoh et al. (73)	Use of Diabetes Pilot™ software on PalmOS® device; recording and monitoring blood glucose, blood pressure, medication usage, physical activity, dietary intake; daily data entry encouraged; Food database; chart report.	12	9.2 ± 1.4	8.1 ± 1.4	9.2 ± 1.6	8.5 ± 1.6
	Franc et al. (52)	Mobile app for self-management; patient enters glycemia, physical activity, and ingested carbohydrates; App calculates insulin dose; automatic data transmission every 2 h; Nightly analytical messages.	12	9.1 ± 1.1	8.69 ± 1.1	9.1 ± 1.0	8.9 ± 1.0

All the incorporated studies were RCT, with a predominant emphasis on diabetes health management based on Apps. The included studies reported a mean duration of diabetes ranging from 0.25 to 17.8 years. This review comprised 5,869 subjects from 41 studies, with a sample size ranging from 22 to 452. The mean (SD) age of participants ranged from 10.4 (1.1) to 69.7 (10.2) years. This review’s App-based intervention focuses on several key areas, such as physical activity, dietary advice, emotional management, medication guidance, and other related aspects. The follow-up time of the intervention ranged from 3 to 24 months, with nine studies having a follow-up period exceeding 12 months. All included studies measured HbA1c outcomes at the baseline and endpoint in both the intervention and control groups.

### 3.2 Primary outcome

**Figure 2:**
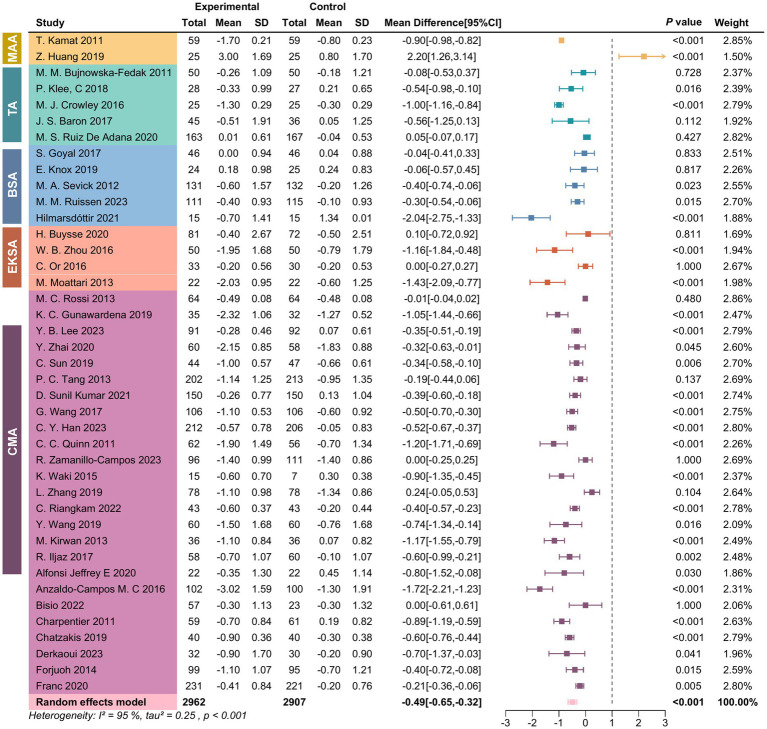
Forest plot illustrating the effect of mobile health on HbA1c.

**Figure 3:**
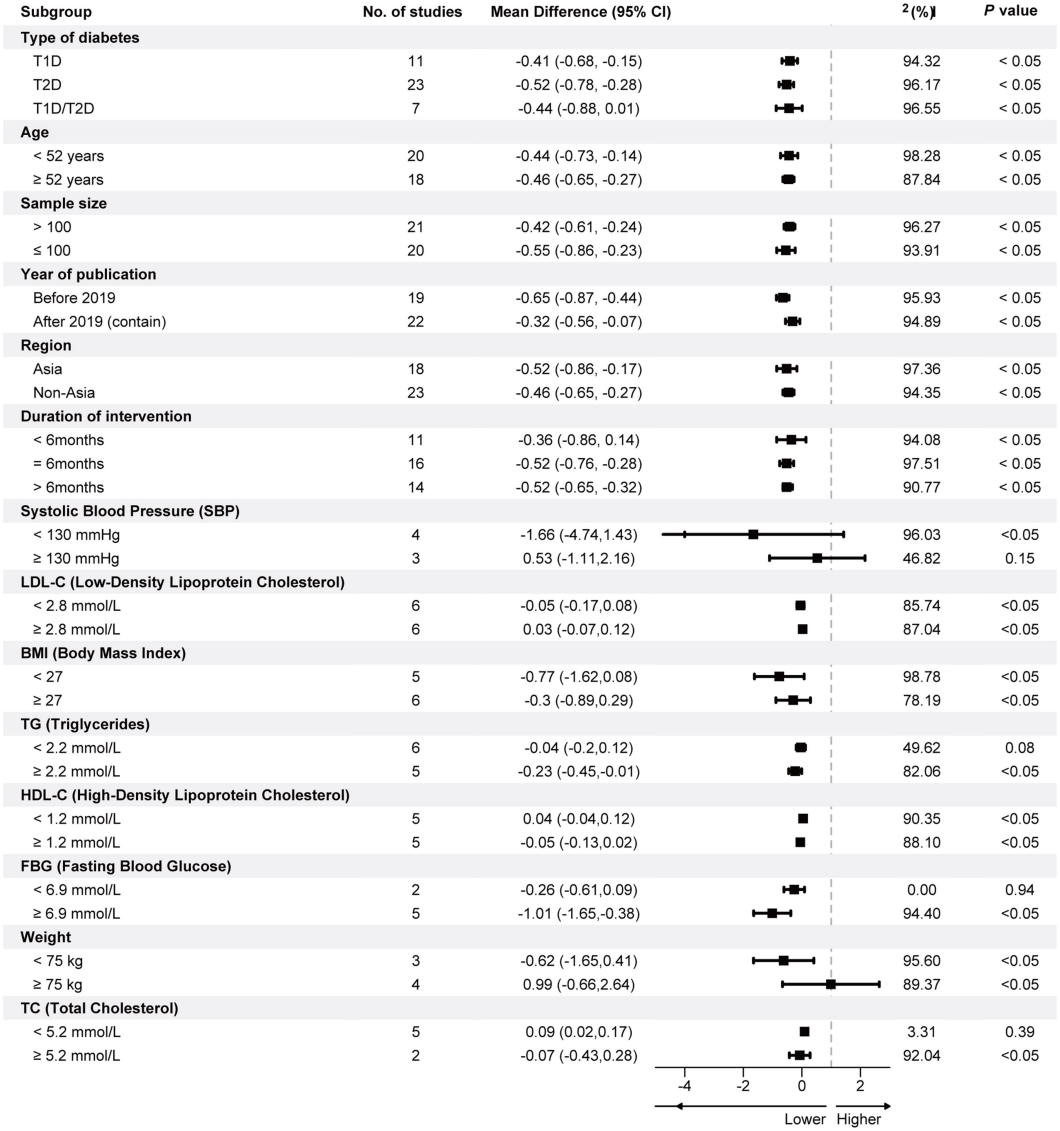
Subgroup analysis forest plot.

Figure 2 shows the summary results in reducing HbA1c among participants with DM. The random effects models showed that mobile phone App strategies were associated with a significant HbA1c reduction by −0.49 (95%CI: −0.65, −0.32) percentage points compared to standard diabetes care. Nonetheless, the studies had considerable heterogeneity in the overall pooled effect (I^2^ = 96.55%, *p* < 0.05). The Subgroup Analysis Forest Plot is presented in Figure 3, showing significant differences in the effects of the intervention across various populations and conditions. Overall, longer intervention durations, specific types of diabetes, and certain physiological subgroups demonstrated more pronounced effects, while some subgroups exhibited high heterogeneity or lacked statistical significance. These findings indicate that the intervention’s effectiveness varies among subgroups, providing a valuable reference for optimizing clinical applications and research design.

#### 3.2.1 Type of diabetes

In 11 studies on T1D patients, the pooled result of the reduction level of HbA1c was −0.41 (95%CI: −0.68, −0.15), and I^2^ (%) was 94.32. For the 23 studies on T2Ds, the pooled result of the reduction level of HbA1c was −0.52 (95%CI: −0.78, −0.28), and I^2^ (%) was 96.17. For the 7 studies on both type 1 and type 2 diabetes, the pooled result of the reduction level of HbA1c was −0.44 (95%CI: −0.88, 0.01), and I^2^ (%) was 96.55.

#### 3.2.2 Age

The age subgroup analysis indicated that participants less than 52 years old had significantly decreased HbA1c levels [−0.44 (95%CI: −0.73, −0.14), I^2^ = 98.28%]. Similarly, patients aged 52 years and older had similar reductions in HbA1c levels [−0.46 (95%CI: −0.65, −0.27), I^2^ = 87.84%].

#### 3.2.3 Sample size

The researchers performed a subgroup analysis based on sample size. The outcomes demonstrated that in the subgroup with a small sample size (less than 100 participants), the overall effect size for HbA1c reduction was statistically significant: −0.55 (95%CI: −0.86, −0.23), I^2^ = 93.91%. For the subgroup with a sample size exceeding 100 participants, the aggregate reduction level of HbA1c is −0.42 (95%CI: −0.61, −0.24), I^2^ = 96.27%.

#### 3.2.4 Year of publication

A subgroup analysis was implemented to understand the variations related to the publication year. The analysis indicated that in the subgroup of studies published earlier than 2019, the reduction in HbA1c was [−0.65 (95%CI: −0.87, −0.44), I^2^ = 95.93%]. On the other hand, for studies published after 2019, the reduction in HbA1c was −0.32 (95%CI: −0.56, −0.07), with high heterogeneity (I^2^ = 94.89%).

#### 3.2.5 Region

A subgroup analysis by region examined the regional differences in the research. The results demonstrated that for the subgroup of the Asia area, the reduction in HbA1c was −0.52 (95% CI: −0.86, −0.17), I^2^ = 97.36. For the subgroup of the non-Asia area, the reduction in HbA1c was −0.46 (95% CI: −0.65, −0.27), with high heterogeneity (I^2^ = 94.35%).

#### 3.2.6 Duration of intervention

Of the 41 included studies, 11 had intervention duration shorter than 6 months, with a pooled HbA1c reduction level of −0.36 (95%CI: −0.86, 0.14). Sixteen studies had an intervention duration of 6 months and had a pooled reduction in HbA1c levels of −0.52 (95%CI: −0.76, −0.28). The remaining 14 studies with intervention duration longer than 6 months had a similar aggregate reduction in HbA1c of −0.52 (95%CI: −0.65, −0.32) compared to 6 months. There was significant heterogeneity in all the above three groups, with the I^2^ being more significant than 90%.

#### 3.2.7 Bubble chart analysis of intervention types and time trends

**Figure 4:**
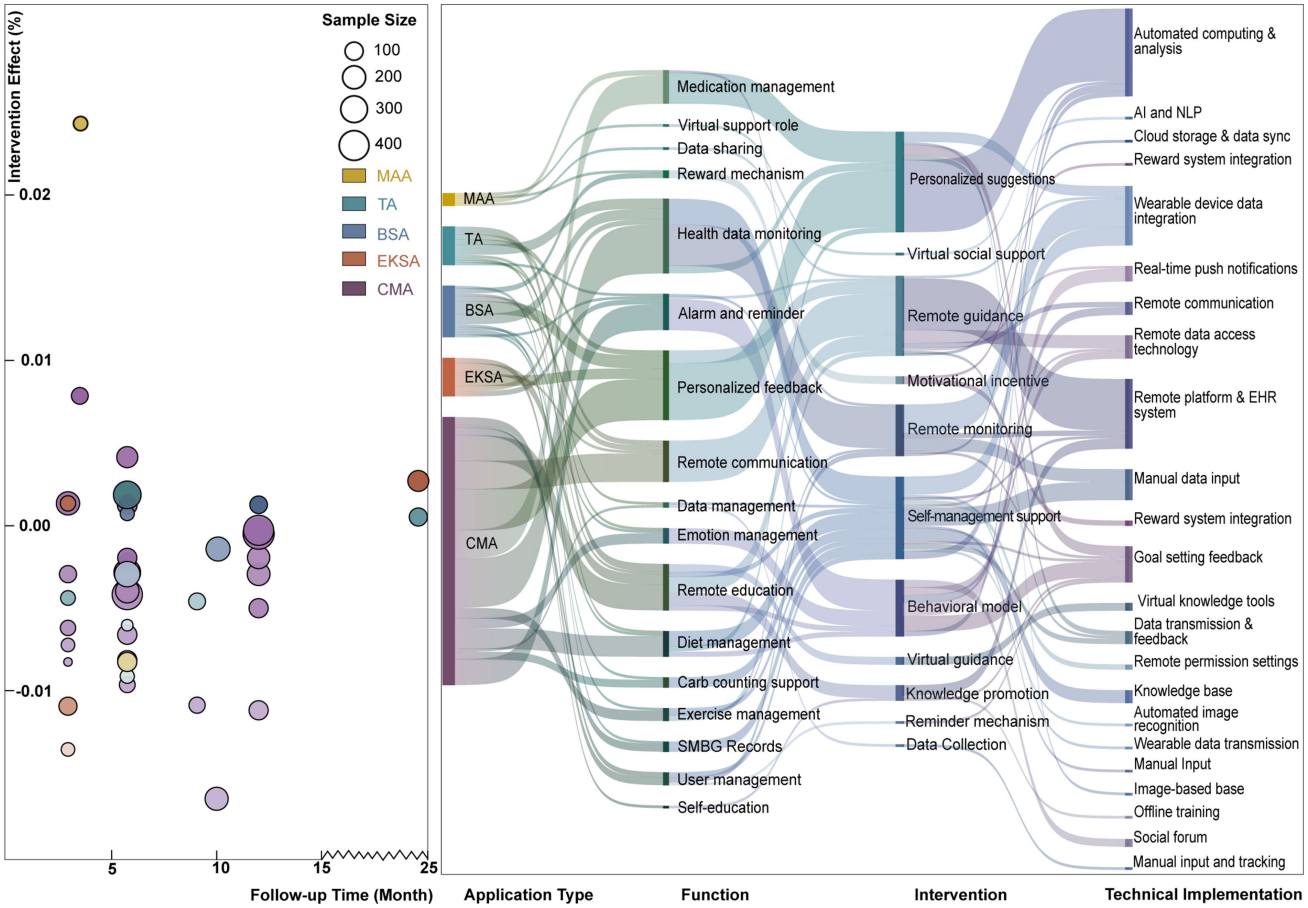
Left: Intervention effect vs. follow-up time across different application types. Right: mapping of application types to functions, interventions, and technical implementations.

Figure 4 evaluates the effectiveness of different mHealth interventions in blood glucose control, analyzing the relationship between intervention types and time trends. Most applications showed a neutral or insignificant effect on blood glucose control. However, studies with larger sample sizes demonstrated higher statistical power. Researchers found that Comprehensive Management Applications (CMA) and Education and Knowledge Support Applications (EKSA) were more effective in reducing HbA1c levels, suggesting that these intervention types could be key to optimizing glycemic management.

Further research into the effectiveness of CMA and EKSA and their potential applications in clinical treatment requires exploration. For interventions with less significant effects, such as Medication Adhesion Applications (MAA), larger-scale clinical trials are suggested to assess their practical impact. Additionally, future study designs should consider the potential influence of follow-up duration on intervention outcomes to enhance the effectiveness of mHealth applications in diabetes management.

#### 3.2.8 Sankey diagram analysis

The flow of different mHealth intervention types is illustrated in Figure 4 using a Sankey diagram, highlighting their primary functions and associated technologies. Medication Adherence Applications (MAA) are primarily directed toward medication management and virtual support roles through automated calculation, analysis, and cloud storage with data synchronization technologies, demonstrating the potential to improve patient medication adherence. TA (Telemedicine Applications) are focused on health data monitoring, relying on remote data access technologies and platform support, and are associated with remote monitoring and guidance interventions. Behavior Support Applications (BSA) are directed toward reminders and personalized feedback, requiring real-time push notifications and remote communication technologies. They are related to motivational incentives and self-management support interventions. Education and Knowledge Support Applications (EKSA) focus on remote education and data management, leverage automated image recognition and virtual knowledge tools, and link to knowledge dissemination and reminder-based interventions. Comprehensive Management Applications (CMA), with their wide coverage of functionalities such as diet and exercise management, rely on integrated technologies and are associated with remote guidance and self-management support interventions.

Due to its broad functionality, CMA is the most effective tool for providing comprehensive health management support. All application types rely on integrated emerging technologies (e.g., AIGC). Given CMA’s potential for delivering holistic health management support, we recommend further studies to evaluate its effectiveness and acceptability across different patient populations.

### 3.3 Quality assessment

**Figure 5:**
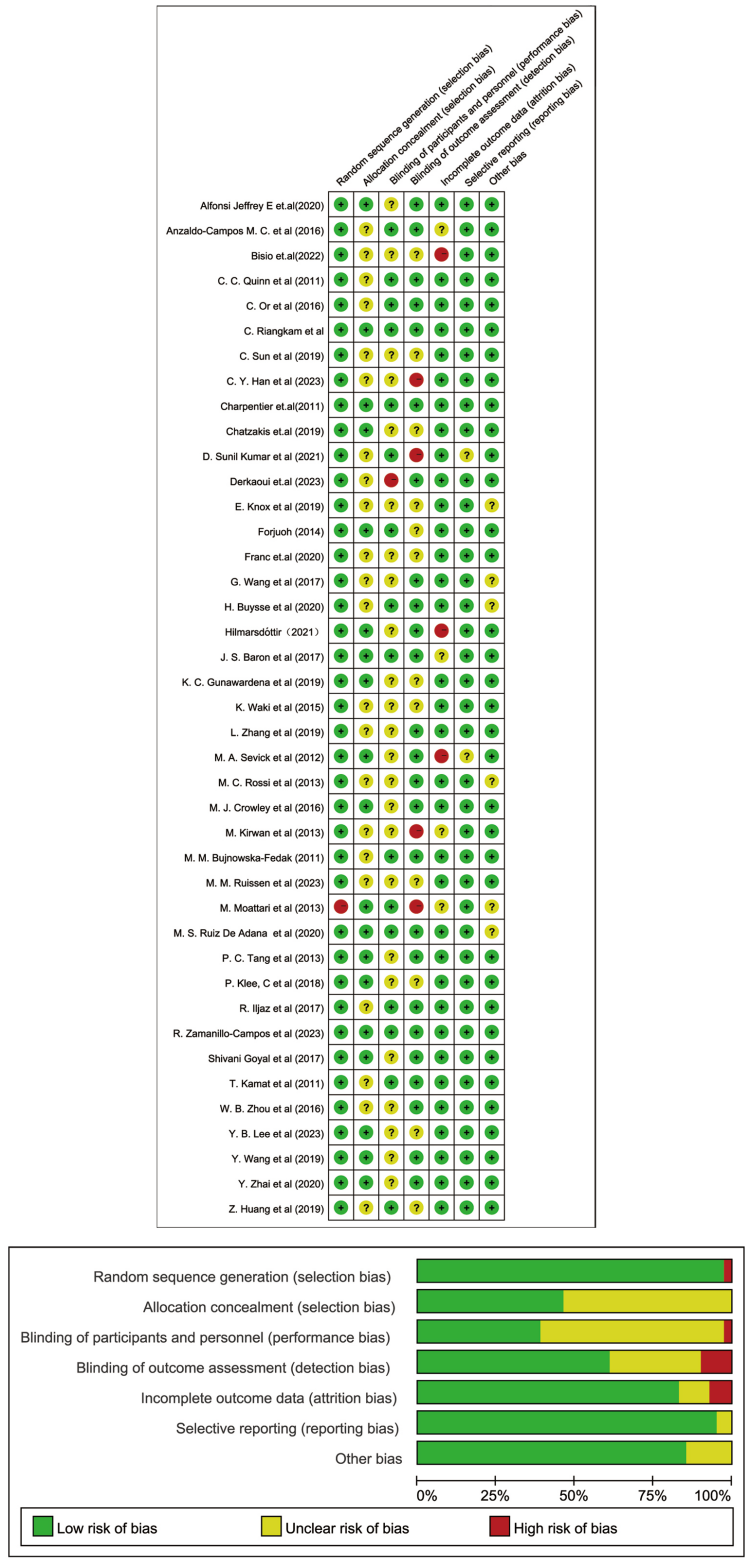
Cochrane risk of bias summary.

The researchers appraised the quality assessment in the 41 included studies using Cochrane Collaboration’s risk of bias tool, as displayed in Figure 5. Among the included studies, 97% demonstrated a low risk of bias in random sequence generation. However, allocation concealment and blinding of participants and personnel were at unclear risk of bias in nearly 60% of the studies. Regarding outcome assessment, except for 10% of the studies with high-risk bias, most other studies had low or uncertain risk bias. Researchers ensured that over 80% of the studies avoided incomplete outcome data, selective reporting, and other biases. Most of the included RCTs demonstrated a low risk of bias across various domains. However, some studies still had some areas of concern that could affect the results’ validity and generalizability.

### 3.4 Publication bias

**Figure 6:**
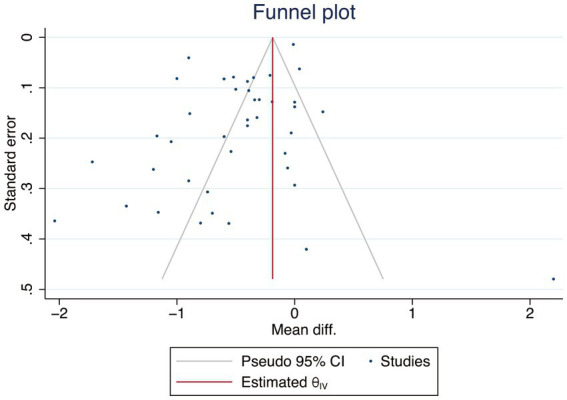
Funnel plot of included studies.

As displayed in Figure 6, the funnel plot was asymmetrical, and Egger’s test yielded a *p*-value of 0.002, suggesting the presence of potential publication bias in the included studies.

## 4 Discussion

This analysis reports on the effects of mobile health (mHealth) management on glycemic control in the two major types of diabetes, type 1 diabetes (T1D) and type 2 diabetes (T2D). Across all included studies, we observed an association between mobile-based health management and reduced HbA1c levels in diabetes patients. mHealth technologies have significantly enhanced the accessibility of healthcare in terms of geography, time, and cost while also improving the efficiency of healthcare professionals. Research shows that, in addition to providing information and educating participants on the importance of understanding their health status and making lifestyle changes, mHealth has markedly improved medication adherence (6, 27–29). mHealth technologies enable patients to send symptom images via mobile devices and engage in remote communication with healthcare providers, reducing unnecessary travel and medical expenses and expanding healthcare coverage. These technologies are particularly beneficial in providing timely medical support for patients in remote areas or with mobility limitations (11, 27, 30). Subgroup analyses confirmed similar effects across different study settings. However, it is important to acknowledge the significant heterogeneity observed among these studies. This analysis also identifies several key challenges and potential directions for optimizing mobile applications in diabetes management.

Most studies show that diabetes management Apps offer a variety of features, including blood glucose monitoring, physical activity instruction, dietary recording, medication reminders, and educational resources. The integration of these features markedly improves patients’ self-management abilities. Regular blood glucose monitoring and data recording help patients understand glucose patterns, adjust lifestyle choices, and modify medication use, thus optimizing blood glucose control (31–34). For example, in the “EMPOWER-D” study, participants could record diabetes-relevant data through an online platform, which included details on dietary habits, physical exercise, home blood pressure measurements, insulin administration, and body weight. These interactive visual representations of the data enabled participants to monitor their progress toward set objectives and to correlate blood glucose levels with adherence to medication regimens or lifestyle modifications (35). Moreover, reminder and notification features are essential for promoting adherence. In particular, medication reminder functions ensure timely medication adherence, reducing the risk of missed doses, which is crucial for diabetes management, and ensuring patients never forget their important health tasks (36–38). Despite these benefits, the variability in the effectiveness of different Apps reflects differences in design, functionality, and user engagement. Future research should focus on refining App features to enhance their overall efficacy. In mobile app-based diabetes health management, the type of diabetes is a crucial factor that affects prognosis. Previous studies have reported that T1D and T2D differ in pathophysiology, management needs, and intervention responses (29, 30, 39). According to the findings of this study, T2D patients may benefit more from mobile interventions due to their typically more manageable condition than T1D. T1D typically requires meticulous insulin management and glucose monitoring, whereas T2D management often focuses more on lifestyle changes and long-term self-management (40, 41). The differential impact of mobile Apps based on diabetes type highlights the importance of tailoring interventions to address specific needs. Apps designed with features and content that align with the distinct requirements of T1D versus T2D will likely result in more effective management outcomes. Future research should continue to explore these differences to refine App functionalities and improve their impact across diverse patient populations (42).

Regional diversity and variations in technology penetration significantly influence the feasibility and effectiveness of mobile health (mHealth) applications. In Asian countries such as India (27), China (33, 34, 43, 44), South Korea (45), and Sri Lanka (36), disparities in smartphone and internet penetration are shaped by regional, educational, and economic differences. For instance, in South Korea, where smartphone penetration is high, technology acceptance is relatively better (45). However, in rural or remote areas with lower educational levels (27, 33, 34, 44, 46), smartphone applications with reminders and reward mechanisms improved patient medication adherence. This improvement was sustained even after 3 months, suggesting that the reward mechanisms might align closely with cultural incentives (47). Conversely, regions in India, Sri Lanka, and parts of China face challenges such as low smartphone penetration and limited patient technological literacy. These barriers may hinder initial usage, especially for older adults and individuals with low technological literacy (34, 43, 44). While technology acceptance is generally higher, regional disparities remain notable in Europe and North America (35, 47–52). For example, a study from Poland highlighted the role of internet penetration in accessing health-related information (48), particularly in remote areas where the availability of technology and patients’ educational backgrounds directly impacted intervention outcomes. Research from Switzerland focused on design challenges for adolescents and children (47), suggesting the simplification of interfaces and the incorporation of gamification to increase engagement, emphasizing cultural and age-specific adaptations (47). Moreover, technological barriers remain significant in certain regions. For instance, populations in remote areas or those with lower socioeconomic status often face challenges such as limited access to smart devices, inadequate network coverage, and low levels of digital literacy (34, 43, 44), making even basic installation and continuous updates of applications highly challenging. In environments with weaker technological infrastructure, data security and privacy concerns are often exacerbated. Patients may lack sufficient understanding of mobile device permissions, data transmission processes, and confidentiality mechanisms, which can reduce their willingness to use these technologies (31, 33, 47, 48). Therefore, merely iterating application functionalities is insufficient to overcome these technical shortcomings. A comprehensive approach is required, involving improvements to infrastructure, widespread promotion of digital health education, and strategic investments at the policy level. Such measures are essential to enhancing the accessibility and efficiency of mHealth on a broader scale (51, 52). Future research should systematically evaluate the root causes of technological barriers and collaborate with key stakeholders, including local governments, healthcare institutions, and mobile network operators, to develop a “technology-service-policy” integrated framework. This approach could provide diverse populations with more stable, user-friendly, and secure digital health management environments (43, 44).

Cultural and social factors profoundly affect the adoption and user experience of mHealth applications. Factors such as patients’ educational levels, religious beliefs, occupations, and attitudes toward health management can significantly influence mHealth acceptance and long-term adherence (27, 31, 36, 45, 53). In environments where family or community support is central, such support significantly enhances user retention. Among multilingual or multiethnic populations, cultural adaptations—including localized language versions and dietary data—are critical (31, 34, 52, 53). However, superficial localization efforts, such as language translation and dietary adjustments, cannot eliminate cultural barriers. Certain social groups may hold conservative or skeptical attitudes toward mobile health (mHealth) technologies, particularly in regions lacking trust in digital innovations and healthcare institutions. These populations often rely more heavily on traditional care methods or health advice from family and friends (34, 43). Furthermore, varying beliefs and values shape perceptions of disease management and personal responsibility, leading to differences in mHealth adoption and adherence. In some cultural contexts, excessive reliance on “external assistance” or discomfort with high-tech interventions may reduce the frequency of use and adherence to mHealth applications (33, 50). Without a thorough understanding of these cultural factors during the design and implementation phases, achieving meaningful behavioral changes or health benefits can be challenging. Future research should systematically account for multi-layered cultural factors and collaborate with local health authorities and community leaders to develop targeted intervention strategies and educational programs to address this. Such efforts are essential for effectively promoting mHealth technologies and ensuring their long-term impact across diverse cultural contexts (33, 51). Recommendations for region-specific modifications suggest that localized reward mechanisms (e.g., points systems, medication discounts) and optimized application interfaces and features (e.g., automated data uploads, streamlined operations) are essential to lowering usage barriers (27, 31, 47, 50). Some studies have utilized behavior change models, such as the Transtheoretical Model, to motivate participants by tailoring content based on language, education level, or ethnicity (38, 53). Action plans generated within applications encourage patients to take responsibility for managing their health and promote behavior change. These plans have proven effective in improving medication adherence and routine self-monitoring, such as reminding patients to adjust insulin doses or monitor blood pressure (27, 45). However, the frequency and personalization of such content require optimization. In some cultural contexts, patients show lower acceptance of message-based services, which may limit intervention effectiveness (40). Future research should prioritize localization and cultural adaptation strategies (50, 51). Researchers should develop differentiated interventions for populations with diverse socioeconomic and cultural backgrounds. Additionally, multicenter and long-term randomized clinical trials are needed to validate further interventions’ effectiveness and scalability (33, 45, 47, 51). Such efforts are essential to driving the global adoption and optimization of mHealth applications.

Analysis of the observed high heterogeneity revealed several potential reasons for significant differences among studies, including the diversity of application functionalities, patient demographics, and differences in study design. First, regarding application functionalities, different types of mobile health applications—such as comprehensive management applications (CMAs), behavioral support applications, and medication adherence applications—exhibited significant differences in their intervention goals and functional implementations. Comprehensive management applications (CMAs) and education and knowledge support applications (EKSAs) demonstrated higher effectiveness, whereas applications focused solely on medication adherence showed weaker effects on glycemic control, as illustrated in Figure 3 (I^2^ = 94.32%). Additionally, the depth of technology use, such as automated analytics, remote monitoring, and real-time feedback, may have further amplified the impact of functional differences on outcomes. Second, the diversity in patient demographics may significantly influence intervention effectiveness. For instance, patients with different diabetes types, ages, education levels, and regional backgrounds exhibit varying technology acceptance, adherence, and usage patterns. For example, the pooled HbA1c improvement for type 1 diabetes patients was −0.41 (I^2^ = 94.32%), for type 2 diabetes patients was −0.52 (I^2^ = 96.17%), and for combined type 1 and type 2 diabetes patients was −0.44 (I^2^ = 96.55%). Additionally, age subgroup analysis (below 52 years: I^2^ = 98.28%; 52 years and above: I^2^ = 87.84%) revealed differences in intervention effects across age groups. Third, heterogeneity in study designs further exacerbated inconsistencies in overall results. Factors such as intervention duration, sample size, follow-up periods, and the quality of bias control varied widely. Studies with longer intervention durations generally reported better outcomes than those with shorter durations. In contrast, studies with larger sample sizes demonstrated more substantial statistical power than those with smaller samples. Subgroup analyses also revealed differences in outcomes based on region (Asian studies: I^2^ = 97.36%; non-Asian studies: I^2^ = 94.35%) and publication period (before 2019: I^2^ = 95.93%; after 2019: I^2^ = 94.89%). These differences likely result from technological advancements, healthcare resources, and cultural contexts. The sources of high heterogeneity highlight the need for future research to adopt more standardized designs, fully consider variations in patient and application characteristics, and employ standardized methods to reduce result inconsistencies. Moreover, larger-scale clinical trials are needed to validate the real-world effectiveness of different intervention types.

Research literature has highlighted the effects of mHealth interventions on diabetes complications, such as retinopathy, kidney damage, cardiovascular events, and long-term health metrics. Studies have associated these interventions with reduced incidence rates of microvascular complications, myocardial infarction, and diabetes-related mortality (34, 43, 44, 54–57). Additionally, many studies have addressed psychosocial aspects, such as “fear of hypoglycemia,” depression, and anxiety, finding that mHealth interventions did not impose additional psychological burdens. In some cases, these interventions even alleviated the fear of hypoglycemia and improved patients’ quality of life (49, 51, 58–60). Studies have emphasized the potential value of sustained glycemic improvements in reducing complication risks and called for future research with more extended follow-up periods and more comprehensive outcome metrics. These should include blood pressure, lipid profiles, mental health, and cardiovascular event rates to evaluate the multidimensional impacts of mHealth interventions (34, 61). Although current research has preliminarily demonstrated benefits beyond glycemic control, researchers must focus on and validate findings for other complications and long-term health outcomes (62–64). The duration of intervention is a critical factor in determining its efficacy. Longer interventions allow patients to adapt to the app, develop good management habits, and achieve more significant health outcomes (48, 49). In contrast, short-term interventions may only partially utilize the app’s functionalities or lead to limited improvements in health status (31, 47, 56). Extending the intervention duration can enhance app usage frequency and adherence while providing continuous feedback and support, reinforcing behavior change. As such, intervention duration is crucial for maximizing the advantages of mobile applications in diabetes management. More extended intervention periods are generally associated with more sustained effects (57, 65).

A few studies focus on personal health records (PHRs) or cloud-sharing platforms (27, 32, 33, 54). However, many studies have explicitly reported cases of technical integration between mobile intervention tools and EHRs. For example, “DiabeText” integrates patient-generated data into existing EHR systems to enable personalized management (28, 34, 35, 47, 59). While some studies conducted within established healthcare networks or remote monitoring systems have demonstrated the overall benefits of EHR-based diabetes management, they lack detailed discussions on interoperability (28, 56). For healthcare providers, EHRs enable flexible scheduling of care services, saving time and improving service quality. They provide a coordinated platform that enhances teamwork, promotes consistent and high-quality care, and supports continuity of care. mHealth fosters collaboration within teams and across institutions by sharing patient information, thereby improving healthcare delivery’s overall quality and efficiency. Feedback from care providers highlights that patients can directly contact their assigned nurses, increasing communication efficiency, optimizing resource utilization, and enhancing productivity (28, 34). Current research primarily focuses on evaluating the effectiveness of mHealth applications, with limited systematic exploration of how to achieve seamless integration between mHealth and EHRs. Critical issues such as ensuring information security, standardizing data interfaces, and enabling clinicians to access and utilize real-time patient-generated data remain underexplored (66, 67). Future studies should delve deeper into integrating mHealth and EHRs, addressing the bidirectional flow of clinical and self-monitoring data. Such efforts are crucial for advancing personalized care and comprehensive healthcare management (28, 35).

Studies have explored the potential of “AI-driven personalized features” in diabetes management. For example, image recognition technologies such as FoodLog (65) and the iSpy application (68) help patients automatically log or analyze dietary intake, effectively reducing the cognitive burden of manual data entry. AI-enabled directly observed therapy (DOT) enhances the accuracy of medication adherence monitoring (27). Systems like POWER2DM (28), Diabeo (57), and Euglyca (69) employ personalized algorithms to assist with insulin dose calculations or provide targeted intervention recommendations based on real-time blood glucose trends and lifestyle data. These advancements demonstrate AI’s feasibility and practical value in offering “individualized, real-time” support for diabetes management while fostering personalized care and stronger connections between patients and healthcare providers. Healthcare providers noted that mHealth applications tailored to patient needs enhance trust and security by enabling continuous communication (34, 37, 46, 51, 60, 70). Flexible scheduling offers patients with night shifts or busy schedules a convenient way to express emotions and strengthens the patient-provider relationship, ultimately improving satisfaction and treatment adherence. However, replacing face-to-face interactions with online services may weaken direct communication, potentially affecting care quality. Some providers expressed concerns that the lack of in-person interaction might lead to misunderstandings about patients’ conditions (39).

Additionally, applications with remote monitoring capabilities, including features such as alerts for abnormal readings, have proven more effective in improving clinical parameters by prompting timely interventions (28, 57, 70). While these explorations have shown promising results, most studies remain limited to basic automation and personalized feedback, falling short of demonstrating the potential of multidimensional data integration or advanced deep-learning models in long-term disease management. Future research combining patient behavior characteristics, dynamic blood glucose changes, and lifestyle information to design and evaluate high-level intelligent algorithms could significantly enhance patient engagement, overall health outcomes, and the quality of patient-provider relationships (37, 38, 62, 66, 68).

Integrating gamification elements into interventions has significantly improved patient engagement and adherence, offering stronger behavioral reinforcement than traditional reminders and reinforcement features (27, 47, 61). For example, transforming the monotonous task of medication adherence into engaging in interactive activities improved medication compliance and glycemic control (HbA1c). It maintained a degree of adherence even after the intervention ceased (45, 47). Some studies have highlighted the potential of gamified applications primarily from design or user experience perspectives, especially for specific groups such as adolescents. Incorporating reward systems and challenge mechanisms has been shown to attract users more effectively (27, 47, 61). Future research should focus on designing a comprehensive framework based on behavioral indicators to evaluate the unique contributions of gamification elements to user engagement, health management, and long-term adherence. Additionally, studies should explore the adaptability and acceptance of gamified designs among different demographic groups (61).

Mobile applications can provide additional features such as real-time communication, group chats, instant feedback, and remote monitoring. These functionalities allow healthcare professionals to offer tailored medication adjustments and lifestyle change recommendations by recording and transmitting data on blood glucose, blood pressure, food intake, and physical activity (27, 71). By reminding participants to conduct regular self-monitoring (e.g., blood glucose or blood pressure) and providing optimized medication recommendations (e.g., insulin dose adjustments), these applications help participants make timely interventions, avoid potential complications, and maintain or improve treatment outcomes. Some patients have reported improved behavioral control, enhanced quality of life, and better health outcomes after using mHealth services (37, 61, 70). mHealth provides tools for self-management support and health behavior change (27, 45, 57, 62), enabling patients to access information and better understand their condition conveniently. Features such as trend charts and health data monitoring increase disease awareness, encourage a more proactive outlook, and strengthen self-management skills. Frequent health tracking and data-driven feedback further reinforce this process. Many applications integrate remote monitoring devices, such as accelerometers, food intake sensors, and wearable sensor patches, with Bluetooth technology to achieve efficient data integration and communication, improving intervention precision and user experience (41). Real-time data collection from blood glucose or blood pressure sensors enables applications to send abnormal reading alerts to physicians, facilitating timely interventions. Other features, such as Bluetooth-enabled wearable devices and trend charts, further enhance intervention efficacy (54, 58).

Age is another critical factor that cannot be overlooked. Younger individuals tend to exhibit greater adaptability and proficiency in app-based diabetes management tools. For example, younger patients are more likely to utilize real-time monitoring, interactive education modules, and social sharing components to enhance their disease awareness and self-management skills (55). In contrast, older patients face unique challenges. As age increases, vision, cognitive processing speed, and familiarity with technology tend to decline, potentially hindering seamless interaction with applications (72).

Additionally, device malfunctions, poorly designed user interfaces, and low data interoperability can reduce patient satisfaction (51, 59, 67). Some patients, particularly older ones, may discontinue use due to technological difficulties or unfamiliarity, underscoring the need to improve patient engagement and adherence (28, 31, 33, 61). The primary barriers for older adults using mHealth applications include low familiarity with smartphones and related technologies, limited digital literacy, and the complexity of interface operations, which may lead to a steep learning curve (28, 31, 33, 48, 61, 67, 72). However, the literature broadly agrees that these barriers are not insurmountable. With the proliferation of technology, simplified interface designs, and enhanced training and support measures, the potential for older adults to adopt mHealth applications remains promising (28, 61, 67). Several studies have highlighted strategies to improve the user experience and adherence of older patients, such as optimizing interface designs (e.g., larger fonts and fewer operational steps), introducing automated data entry functions (e.g., automatic synchronization with glucometers), and providing continuous remote support (48, 49, 61, 72). For instance, research has shown that after an adaptation period of 3 to 6 months with remote guidance, older patients gradually mastered using mHealth applications and achieved significant improvements in health metrics such as glycemic control (48, 61). Other studies have emphasized the need for more personalized and user-friendly designs for older users, such as reducing manual data input and enhancing real-time feedback features to lower technological barriers (31, 33, 48, 61). Despite these advancements, most studies have not profoundly explored the specific barriers older populations face or the corresponding solutions. Future research should focus on digital health literacy and long-term usage behaviors of older adults, designing more inclusive systems to serve this important demographic better and improve health management outcomes (31, 61, 72).

In summary, factors such as diabetes type, age, geographic region, and intervention duration significantly influence the effectiveness of app-based diabetes interventions. Future efforts should optimize the design and functionality of mobile health (mHealth) technologies to address the characteristics and needs of diverse patient populations, thereby enhancing the overall effectiveness of diabetes management and improving patients’ quality of life.

While mHealth interventions offer notable advantages, such as improving healthcare accessibility and empowering patients with self-management capabilities, they also face significant technical and usability challenges. Patients, particularly older adults and those with lower education levels may struggle with technological barriers due to limited digital literacy. Issues like device malfunction, unstable networks, and poor data interoperability further constrain the practical utility of these interventions. Additionally, concerns around data privacy breaches, the absence of standardized procedures, and unclear divisions of responsibility may introduce legal and ethical dilemmas. Furthermore, mHealth can increase healthcare providers’ workloads, as additional time and effort are required for patient education and data monitoring, potentially disrupting regular workflows. The reduced face-to-face interaction between patients and providers may also limit nonverbal communication and emotional engagement, potentially hindering the development of deeper patient-provider relationships.

The strengths of this meta-analysis include its broad research sample, encompassing studies conducted across various regions and healthcare settings, as well as data from diabetic patients of different ages and disease durations, thereby enhancing the generalizability and applicability of the findings. Rigorous data analysis methods were employed, adhering strictly to systematic review methodologies, ensuring the study’s scientific validity, consistency, and reliability.

Several limitations of this study warrant consideration. First, significant heterogeneity among existing studies may affect the stability and generalizability of the results. Second, while some studies demonstrated high methodological quality, others exhibited limitations, such as small sample sizes or short follow-up periods, which may undermine the accuracy of the findings. Funnel plot analysis and Egger’s test suggest the presence of publication bias, which may overestimate the observed effects or overlook negative results. Despite efforts to include gray literature, incomplete disclosure of information or missing data for quality assessment prevented the inclusion of certain studies. Moreover, critical research data from companies or institutions may remain undisclosed due to commercial confidentiality or intellectual property concerns, further exacerbating publication bias. This tendency to publish positive findings while internalizing negative or nonsignificant results highlights the need for improved transparency. Expanding data access and fostering collaborations with industry stakeholders and non-public platforms may mitigate these biases in future research.

Future research should focus on increasing sample sizes, extending follow-up durations, and evaluating the effectiveness of mHealth interventions across diverse cultural and healthcare systems. Leveraging advanced technologies such as artificial intelligence and the Internet of Things can enhance the precision of data analysis and intervention strategies while strengthening privacy protections and advancing standardization. Addressing the needs of specific patient populations, particularly through adaptive designs tailored for older adults, will be key to driving the further development of mHealth technologies in diabetes management.

## 5 Conclusion

This review provides a comprehensive summary of the existing evidence on mobile application-based interventions in diabetes management. The findings indicate that these tools offer patients convenient services such as blood glucose monitoring, dietary planning, exercise guidance, and health education, significantly enhancing patients’ self-management awareness and capabilities. However, several practical challenges exist in clinical practice, including technical issues, user acceptance, data security and privacy protection, variations in digital literacy, and adaptability to different cultural and economic contexts. These barriers have limited the widespread adoption and sustainability of these interventions. Furthermore, it is essential to acknowledge the limitations of this study and the included literature, such as variability in study design and quality, publication bias, and heterogeneity, which may affect the stability and generalizability of the results.

In light of these findings, future research and practice should not only focus on the sustained impact of these tools on diabetes management and their potential to improve patient’s quality of life but also aim to optimize intervention design, enhance technical and privacy safeguards, and address potential publication and selection biases in study design. To improve external validity and generalizability, larger-scale, multicenter, and long-term randomized controlled trials should include more diverse populations and settings, particularly vulnerable groups such as the older adult, low-income populations, and ethnic minorities, to evaluate the real-world effectiveness of these interventions in different cultural and social contexts. Additionally, strengthening digital literacy education and refining legal and regulatory frameworks can effectively mitigate barriers related to technical concerns and privacy issues. As mobile technologies continue to evolve, these improvements will help unlock the full potential of digital health management in diabetes care, ultimately leading to significant improvements in patient’s health outcomes and quality of life.

## Data Availability

The original contributions presented in the study are included in the article/Supplementary material, further inquiries can be directed to the corresponding author.
