# Supplementary material for: Technological functionality and system architecture of mobile health interventions for diabetes management: a systematic review and meta-analysis of randomized controlled trials
Source: Front Public Health. 2025 Feb 20;13:1549568. doi: 10.3389/fpubh.2025.1549568 (PMC11884075; doi:10.3389/fpubh.2025.1549568)
Supplement: Supplementary file 1 [file Table_1.DOCX]

Supplementary Material

# Search strings

MBASE

Date of search: 1/11/2024

| # | Search | Results |
| --- | --- | --- |
| 1 | diabetes AND mellitus OR t1dm OR t2dm OR t1d OR t2d OR diabetic* OR prediabetes | 1,417,288 |
| 2 | blood AND sugar OR (hemoglobin AND a1c) OR hba1c OR 'hyperglycemia' | 342,081 |
| 3 | 1 OR 2 | 1,515,835 |
| 4 | continuous AND glucose AND monitoring AND app* OR ('self monitoring' AND of AND blood AND glucose AND app*) OR (mobile AND applications) OR (digital AND health) OR (online AND health) | 408,464 |
| 5 | telerehabilitation OR (medication AND therapy AND management) OR (machine AND learning) OR (artificial AND intelligence) OR (deep AND learning) OR telemedicine | 265,454 |
| 6 | 4 OR 5 | 651,538 |
| 7 | Randomized controlled trial | 1,176,283 |
| 8 | 3 AND 6 AND 7 | 5202 |
| 9 | Limit 8 to yr=”2010-Current” | 3829 |
| 10 | Limit 9 to ('diabetes mellitus'/dm OR 'diabetic ketoacidosis'/dm OR 'diabetic nephropathy'/dm OR 'diabetic neuropathy'/dm OR 'gestational diabetes'/dm OR 'hyperglycemia'/dm OR 'hypoglycemia'/dm OR 'impaired glucose tolerance'/dm OR 'insulin dependent diabetes mellitus'/dm OR 'insulin resistance'/dm OR 'non insulin dependent diabetes mellitus'/dm) | 1509 |

Web of science

Date of search: 1/11/2024

| # | Search | Results |
| --- | --- | --- |
| 1 | Diabetes Mellitus (Topic) or T1DM (Topic) or T2DM (Topic) or T1D (Topic) or T2D (Topic) or diabetic* (Topic) or prediabetes (Topic) | 631,620 |
| 2 | (((((TS=(Blood Sugar)) OR TS=(fasting sugar)) OR TS=(Hemoglobin A1c)) OR TS=(HbA1c)) OR TS=(Hyperglycemia)) OR TS=(Postprandial Hyperglycemias) | 131,124 |
| 3 | 1 OR 2 | 684,447 |
| 4 | (((((((TS=(Continuous Glucose Monitoring app*)) OR TS=(CGM Device)) OR TS=(Self-Monitoring of Blood Glucose app*)) OR TS=(Mobile Applications)) OR TS=(Portable Software Apps)) OR TS=(Portable Electronic App)) OR TS=(digital health)) OR TS=(online health) | 296,813 |
| 5 | (((((((TS=(Telerehabilitation)) OR TS=(Remote Rehabilitation)) OR TS=(Virtual Rehabilitation)) OR TS=(Telemedicine)) OR TS=(Medication Therapy Management)) OR TS=(Machine learning)) OR TS=(Artificial Intelligence)) OR TS=(Deep Learning) | 873,846 |
| 6 | 4 OR 5 | 1,148,605 |
| 7 | TS=(Randomized controlled trial) | 547,021 |
| 8 | 3 AND 6 AND 7 | 1890 |
| 9 | Limit 8 to yr=”2010-Current” | 1431 |
| 10 | Limit 10 to Article (Document Types) | 978 |
| 11 | Limit 9 to English (Languages) | 522 |

Scopus

Date of search: 1/11/2024

| # | Search | Results |
| --- | --- | --- |
| 1 | ( TITLE-ABS-KEY ( prediabetes ) ) OR ( TITLE-ABS-KEY ( diabetic* ) ) OR ( TITLE-ABS-KEY ( t2d ) ) OR ( TITLE-ABS-KEY ( t1d ) ) OR ( TITLE-ABS-KEY ( t2dm ) ) OR ( TITLE-ABS-KEY ( t1dm ) ) OR ( TITLE-ABS-KEY ( diabetes AND mellitus ) ) | 1,043,105 |
| 2 | ( TITLE-ABS-KEY ( postprandial AND hyperglycemias ) ) OR ( TITLE-ABS-KEY ( hyperglycemia ) ) OR ( TITLE-ABS-KEY ( hba1c ) ) OR ( TITLE-ABS-KEY ( hemoglobin AND a1c ) ) OR ( TITLE-ABS-KEY ( fasting AND sugar ) ) OR ( TITLE-ABS-KEY ( blood AND sugar ) ) | 218,431 |
| 3 | 1 OR 2 | 1,139,576 |
| 4 | ( TITLE-ABS-KEY ( online AND health ) ) OR ( TITLE-ABS-KEY ( digital AND health ) ) OR ( TITLE-ABS-KEY ( portable AND electronic AND app ) ) OR ( TITLE-ABS-KEY ( portable AND software AND apps ) ) OR ( TITLE-ABS-KEY ( mobile AND applications ) ) OR ( TITLE-ABS-KEY ( self-monitoring AND of AND blood AND glucose AND app* ) ) OR ( TITLE-ABS-KEY ( cgm AND device ) ) OR ( TITLE-ABS-KEY ( continuous AND glucose AND monitoring AND app* ) ) | 457,838 |
| 5 | ( TITLE-ABS-KEY ( deep AND learning ) ) OR ( TITLE-ABS-KEY ( artificial AND intelligence ) ) OR ( TITLE-ABS-KEY ( machine AND learning ) ) OR ( TITLE-ABS-KEY ( medication AND therapy AND management ) ) OR ( TITLE-ABS-KEY ( telemedicine ) ) OR ( TITLE-ABS-KEY ( virtual AND rehabilitation ) ) OR ( TITLE-ABS-KEY ( telerehabilitation ) ) OR ( TITLE-ABS-KEY ( remote AND rehabilitation ) ) | 826,066 |
| 6 | 4 OR 5 | 1,035,356 |
| 7 | TITLE-ABS-KEY ( randomized AND controlled AND trial ) | 1,079,734 |
| 8 | 3 AND 6 AND 7 | 2016 |
| 9 | Limit 8 to yr=”2010-Current” | 1296 |
| 10 | Limit 9 to English (Languages) | 725 |

PubMed

Date of search: 1/11/2024

| # | Search | Results |
| --- | --- | --- |
| 1 | ((Randomized controlled trial) AND ((((((Continuous Glucose Monitoring app*) OR (Self-Monitoring of Blood Glucose app*)) OR (Mobile Applications)) OR (digital health)) OR (online health)) OR ((((((Telerehabilitation) OR (Telemedicine)) OR (Medication Therapy Management)) OR (Machine learning)) OR (Artificial Intelligence)) OR (Deep Learning)))) AND ((((((((Diabetes Mellitus) OR (T1DM)) OR (T2DM)) OR (T1D)) OR (T2D)) OR (diabetic*)) OR (prediabetes)) OR ((((Blood Sugar) OR (Hemoglobin A1c)) OR (HbA1c)) OR (Hyperglycemia))) | 2077 |
| 2 | Limit 8 to yr=”2010-Current” | 1458 |
| 3 | Limit 9 to English (Languages) | 1138 |

Cochrane

Date of search: 1/11/2024

| # | Search | Results |
| --- | --- | --- |
| 1 | "diabetes mellitus" OR T1DM OR T2DM OR T1D OR T2D OR diabetic* OR prediabetes | 109,329 |
| 2 | "blood sugar" OR "hemoglobin A1c" OR HbA1c OR hyperglycemia | 41817 |
| 3 | 1 OR 2 | 120,187 |
| 4 | (continuous NEXT glucose NEXT monitoring NEXT app*) OR (self-monitoring NEXT of NEXT blood NEXT glucose NEXT app*) OR "mobile applications" OR "digital health" OR "online health" | 4294 |
| 5 | Telerehabilitation OR Telemedicine OR "Medication Therapy Management" OR "Machine learning" OR "Artificial Intelligence" OR "Deep Learning" | 15488 |
| 6 | 4 OR 5 | 18970 |
| 7 | "Randomized Controlled Trial" | 725,962 |
| 8 | 3 AND 6 AND 7 | 5043 |
| 9 | Limit 8 to source PUBMED or EMBASE or CT.gov or ICTRP | 58 |
| 10 | Limit 9 to Content type: Trails | 29 |
| 11 | Limit 10 to publication Year between 2010 and 2024 | 17 |

# The list of the excluded records during the process of full-text checking

| **Not a mobile health application (n=36)** | |
| --- | --- |
| 1 | Cho JH, Kim HS, Yoo SH, et al. An Internet-based health gateway device for interactive communication and automatic data uploading: Clinical efficacy for type 2 diabetes in a multi-centre trial. J Telemed Telecare. 2017;23(6):595-604. doi:10.1177/1357633X16657500 |
| 2 | Lu Z, Li Y, He Y, et al. Internet-Based Medication Management Services Improve Glycated Hemoglobin Levels in Patients with Type 2 Diabetes. Telemed J E Health. 2021;27(6):686-693. doi:10.1089/tmj.2020.0123 |
| 3 | Emerson JF, Welch M, Rossman WE, et al. A Multidisciplinary Intervention Utilizing Virtual Communication Tools to Reduce Health Disparities: A Pilot Randomized Controlled Trial. Int J Environ Res Public Health. 2015;13(1):ijerph13010031. Published 2015 Dec 22. doi:10.3390/ijerph13010031 |
| 4 | Greenwood DA, Blozis SA, Young HM, Nesbitt TS, Quinn CC. Overcoming Clinical Inertia: A Randomized Clinical Trial of a Telehealth Remote Monitoring Intervention Using Paired Glucose Testing in Adults With Type 2 Diabetes. J Med Internet Res. 2015;17(7):e178. Published 2015 Jul 21. doi:10.2196/jmir.4112 |
| 5 | Harashima S, Fukushima T, Sasaki M, et al. Self-monitoring of blood glucose (SMBG) improves glycaemic control in oral hypoglycaemic agent (OHA)-treated type 2 diabetes (SMBG-OHA study). Diabetes Metab Res Rev. 2013;29(1):77-84. doi:10.1002/dmrr.2363 |
| 6 | Jacobs PG, Resalat N, Hilts W, et al. Integrating metabolic expenditure information from wearable fitness sensors into an AI-augmented automated insulin delivery system: a randomised clinical trial. Lancet Digit Health. 2023;5(9):e607-e617. doi:10.1016/S2589-7500(23)00112-7 |
| 7 | Jeong JY, Jeon JH, Bae KH, et al. Smart Care Based on Telemonitoring and Telemedicine for Type 2 Diabetes Care: Multi-Center Randomized Controlled Trial. Telemed J E Health. 2018;24(8):604-613. doi:10.1089/tmj.2017.0203 |
| 8 | Nagrebetsky A, Larsen M, Craven A, et al. Stepwise self-titration of oral glucose-lowering medication using a mobile telephone-based telehealth platform in type 2 diabetes: a feasibility trial in primary care. J Diabetes Sci Technol. 2013;7(1):123-134. Published 2013 Jan 1. doi:10.1177/193229681300700115 |
| 9 | Davis RM, Hitch AD, Salaam MM, Herman WH, Zimmer-Galler IE, Mayer-Davis EJ. TeleHealth improves diabetes self-management in an underserved community: diabetes TeleCare. Diabetes Care. 2010;33(8):1712-1717. doi:10.2337/dc09-1919 |
| 10 | Ballesta S, Chillarón JJ, Inglada Y, et al. Telehealth model versus in-person standard care for persons with type 1 diabetes treated with multiple daily injections: an open-label randomized controlled trial. Front Endocrinol (Lausanne). 2023;14:1176765. Published 2023 Jun 27. doi:10.3389/fendo.2023.1176765 |
| 11 | Vaughan EM, Hyman DJ, Naik AD, Samson SL, Razjouyan J, Foreyt JP. A Telehealth-supported, Integrated care with CHWs, and MEdication-access (TIME) Program for Diabetes Improves HbA1c: a Randomized Clinical Trial. J Gen Intern Med. 2021;36(2):455-463. doi:10.1007/s11606-020-06017-4 |
| 12 | Rasmussen OW, Lauszus FF, Loekke M. Telemedicine compared with standard care in type 2 diabetes mellitus: A randomized trial in an outpatient clinic. J Telemed Telecare. 2016;22(6):363-368. doi:10.1177/1357633X15608984 |
| 13 | Plachy L, Neuman V, Velichova K, et al. Telemedicine maintains good glucose control in children with type 1 diabetes but is not time saving for healthcare professionals: KITES randomized study. Diabetes Res Clin Pract. 2024;209:111602. doi:10.1016/j.diabres.2024.111602 |
| 14 | SuMMiT-D Collaborative Group , Farmer AJ, Allen J, et al. Supporting people with type 2 diabetes in effective use of their medicine through mobile health technology integrated with clinical care (SuMMiT-D pilot): results of a feasibility randomised trial. Pilot Feasibility Stud. 2024;10(1):15. Published 2024 Jan 25. doi:10.1186/s40814-023-01429-5 |
| 15 | Kardas P, Lewandowski K, Bromuri S. Type 2 Diabetes Patients Benefit from the COMODITY12 mHealth System: Results of a Randomised Trial. J Med Syst. 2016;40(12):259. doi:10.1007/s10916-016-0619-x |
| 16 | Kiran S, Nagarajappa VH, Sathyanarayana SO, Hegde A, Raghupathy P. Use of Continuous Glucose Monitoring System in Children with Type 1 Diabetes Mellitus in a Resource Limited Setting. Indian J Endocrinol Metab. 2023;27(3):208-212. doi:10.4103/ijem.ijem_376_22 |
| 17 | Onyia AU, Berhie G, Cecchetti A, Hines A. The Use of Digital Telehealth for the Self-Management of Type 2 Diabetes Patients in Hinds County, Mississippi: A Pilot Study. J Patient Exp. 2023;10:23743735231188835. Published 2023 Oct 3. doi:10.1177/23743735231188835 |
| 18 | Dario C, Toffanin R, Calcaterra F, et al. Telemonitoring of Type 2 Diabetes Mellitus in Italy. Telemed J E Health. 2017;23(2):143-152. doi:10.1089/tmj.2015.0224 |
| 19 | Wang J, Cai C, Padhye N, Orlander P, Zare M. A Behavioral Lifestyle Intervention Enhanced With Multiple-Behavior Self-Monitoring Using Mobile and Connected Tools for Underserved Individuals With Type 2 Diabetes and Comorbid Overweight or Obesity: Pilot Comparative Effectiveness Trial. JMIR Mhealth Uhealth. 2018;6(4):e92. Published 2018 Apr 10. doi:10.2196/mhealth.4478 |
| 20 | Miller KM, Kanapka LG, Rickels MR, et al. Benefit of Continuous Glucose Monitoring in Reducing Hypoglycemia Is Sustained Through 12 Months of Use Among Older Adults with Type 1 Diabetes. Diabetes Technol Ther. 2022;24(6):424-434. doi:10.1089/dia.2021.0503 |
| 21 | McAuley SA, Trawley S, Vogrin S, et al. Closed-Loop Insulin Delivery Versus Sensor-Augmented Pump Therapy in Older Adults With Type 1 Diabetes (ORACL): A Randomized, Crossover Trial. Diabetes Care. 2022;45(2):381-390. doi:10.2337/dc21-1667 |
| 22 | Renard E, Tubiana-Rufi N, Bonnemaison-Gilbert E, et al. Closed-loop driven by control-to-range algorithm outperforms threshold-low-glucose-suspend insulin delivery on glucose control albeit not on nocturnal hypoglycaemia in prepubertal patients with type 1 diabetes in a supervised hotel setting. Diabetes Obes Metab. 2019;21(1):183-187. doi:10.1111/dom.13482 |
| 23 | Nelson LA, Greevy RA, Spieker A, et al. Effects of a Tailored Text Messaging Intervention Among Diverse Adults With Type 2 Diabetes: Evidence From the 15-Month REACH Randomized Controlled Trial. Diabetes Care. 2021;44(1):26-34. doi:10.2337/dc20-0961 |
| 24 | Middleton T, Constantino M, McGill M, et al. An Enhanced SMS Text Message-Based Support and Reminder Program for Young Adults With Type 2 Diabetes (TEXT2U): Randomized Controlled Trial. J Med Internet Res. 2021;23(10):e27263. Published 2021 Oct 21. doi:10.2196/27263 |
| 25 | Nelson LA, Spieker AJ, Mayberry LS, McNaughton C, Greevy RA. Estimating the impact of engagement with digital health interventions on patient outcomes in randomized trials. J Am Med Inform Assoc. 2021;29(1):128-136. doi:10.1093/jamia/ocab254 |
| 26 | Sadanshiv M, Jeyaseelan L, Kirupakaran H, Sonwani V, Sudarsanam TD. Feasibility of computer-generated telephonic message-based follow-up system among healthcare workers with diabetes: a randomized controlled trial. BMJ Open Diabetes Res Care. 2020;8(1):e001237. doi:10.1136/bmjdrc-2020-001237 |
| 27 | Lee J, Lee MH, Park J, et al. FGM-based remote intervention for adults with type 1 diabetes: The FRIEND randomized clinical trial. Front Endocrinol (Lausanne). 2022;13:1054697. Published 2022 Nov 25. doi:10.3389/fendo.2022.1054697 |
| 28 | Billings LK, Parkin CG, Price D. Baseline Glycated Hemoglobin Values Predict the Magnitude of Glycemic Improvement in Patients with Type 1 and Type 2 Diabetes: Subgroup Analyses from the DIAMOND Study Program. Diabetes Technol Ther. 2018;20(8):561-565. doi:10.1089/dia.2018.0163 |
| 29 | Crowley MJ, Tarkington PE, Bosworth HB, et al. Effect of a Comprehensive Telehealth Intervention vs Telemonitoring and Care Coordination in Patients With Persistently Poor Type 2 Diabetes Control: A Randomized Clinical Trial. JAMA Intern Med. 2022;182(9):943-952. doi:10.1001/jamainternmed.2022.2947 |
| 30 | du Pon E, Kleefstra N, Cleveringa F, van Dooren A, Heerdink ER, van Dulmen S. Effects of the Proactive Interdisciplinary Self-Management (PRISMA) Program on Online Care Platform Usage in Patients with Type 2 Diabetes in Primary Care: A Randomized Controlled Trial. J Diabetes Res. 2020;2020:5013142. Published 2020 Jan 8. doi:10.1155/2020/5013142 |
| 31 | Farmer A, Bobrow K, Leon N, et al. Digital messaging to support control for type 2 diabetes (StAR2D): a multicentre randomised controlled trial [published correction appears in BMC Public Health. 2022 Apr 13;22(1):710. doi: 10.1186/s12889-022-13085-0]. BMC Public Health. 2021;21(1):1907. Published 2021 Oct 21. doi:10.1186/s12889-021-11874-7 |
| 32 | Feng Y, Zhao Y, Mao L, et al. The Effectiveness of an eHealth Family-Based Intervention Program in Patients With Uncontrolled Type 2 Diabetes Mellitus (T2DM) in the Community Via WeChat: Randomized Controlled Trial. JMIR Mhealth Uhealth. 2023;11:e40420. Published 2023 Mar 20. doi:10.2196/40420 |
| 33 | Fortmann AL, Gallo LC, Garcia MI, et al. Dulce Digital: An mHealth SMS-Based Intervention Improves Glycemic Control in Hispanics With Type 2 Diabetes. Diabetes Care. 2017;40(10):1349-1355. doi:10.2337/dc17-0230 |
| 34 | Fountoulakis S, Papanastasiou L, Gryparis A, Markou A, Piaditis G. Impact and duration effect of telemonitoring on ΗbA1c, BMI and cost in insulin-treated Diabetes Mellitus patients with inadequate glycemic control: A randomized controlled study. Hormones (Athens). 2015;14(4):632-643. doi:10.14310/horm.2002.1603 |
| 35 | Hansel B, Giral P, Gambotti L, et al. A Fully Automated Web-Based Program Improves Lifestyle Habits and HbA1c in Patients With Type 2 Diabetes and Abdominal Obesity: Randomized Trial of Patient E-Coaching Nutritional Support (The ANODE Study). J Med Internet Res. 2017;19(11):e360. Published 2017 Nov 8. doi:10.2196/jmir.7947 |
| 36 | Hu L, Shi Y, Wylie-Rosett J, et al. Feasibility of a family-oriented mHealth intervention for Chinese Americans with type 2 diabetes: A pilot randomized control trial. PLoS One. 2024;19(3):e0299799. Published 2024 Mar 11. doi:10.1371/journal.pone.0299799 |
| **Intervention not focused on patient self-management (n=35)** | |
| 1 | Dong Y, Wang P, Dai Z, et al. Increased self-care activities and glycemic control rate in relation to health education via Wechat among diabetes patients: A randomized clinical trial. Medicine (Baltimore). 2018;97(50):e13632. doi:10.1097/MD.0000000000013632 |
| 2 | Ye H, Lin L, Zhong D, et al. The impact of telehealth education on self-management in patients with coexisting type 2 diabetes mellitus and hypertension: a 26-week randomized controlled trial. J Endocrinol Invest. 2024;47(9):2361-2369. doi:10.1007/s40618-024-02310-9 |
| 3 | Bell AM, Fonda SJ, Walker MS, Schmidt V, Vigersky RA. Mobile phone-based video messages for diabetes self-care support. J Diabetes Sci Technol. 2012;6(2):310-319. Published 2012 Mar 1. doi:10.1177/193229681200600214 |
| 4 | Bollyky JB, Bravata D, Yang J, Williamson M, Schneider J. Remote Lifestyle Coaching Plus a Connected Glucose Meter with Certified Diabetes Educator Support Improves Glucose and Weight Loss for People with Type 2 Diabetes. J Diabetes Res. 2018;2018:3961730. Published 2018 May 16. doi:10.1155/2018/3961730 |
| 5 | Christensen JR, Laursen DH, Lauridsen JT, et al. Reversing Type 2 Diabetes in a Primary Care-Anchored eHealth Lifestyle Coaching Programme in Denmark: A Randomised Controlled Trial. Nutrients. 2022;14(16):3424. Published 2022 Aug 19. doi:10.3390/nu14163424 |
| 6 | Kotsani K, Antonopoulou V, Kountouri A, et al. The role of telenursing in the management of Diabetes Type 1: A randomized controlled trial. Int J Nurs Stud. 2018;80:29-35. doi:10.1016/j.ijnurstu.2018.01.003 |
| 7 | Liou JK, Soon MS, Chen CH, et al. Shared care combined with telecare improves glycemic control of diabetic patients in a rural underserved community. Telemed J E Health. 2014;20(2):175-178. doi:10.1089/tmj.2013.0037 |
| 8 | Nanditha A, Thomson H, Susairaj P, et al. A pragmatic and scalable strategy using mobile technology to promote sustained lifestyle changes to prevent type 2 diabetes in India and the UK: a randomised controlled trial. Diabetologia. 2020;63(3):486-496. doi:10.1007/s00125-019-05061-y |
| 9 | Ramadas A, Chan CKY, Oldenburg B, Hussein Z, Quek KF. Randomised-controlled trial of a web-based dietary intervention for patients with type 2 diabetes: changes in health cognitions and glycemic control. BMC Public Health. 2018;18(1):716. Published 2018 Jun 8. doi:10.1186/s12889-018-5640-1 |
| 10 | Yaron M, Sher B, Sorek D, et al. A randomized controlled trial comparing a telemedicine therapeutic intervention with routine care in adults with type 1 diabetes mellitus treated by insulin pumps. Acta Diabetol. 2019;56(6):667-673. doi:10.1007/s00592-019-01300-1 |
| 11 | Carolan-Olah M, Sayakhot P. A randomized controlled trial of a web-based education intervention for women with gestational diabetes mellitus. Midwifery. 2019;68:39-47. doi:10.1016/j.midw.2018.08.019 |
| 12 | Asante E, Bam V, Diji AK, et al. Pilot Mobile Phone Intervention in Promoting Type 2 Diabetes Management in an Urban Area in Ghana: A Randomized Controlled Trial. Diabetes Educ. 2020;46(5):455-464. doi:10.1177/0145721720954070 |
| 13 | Kim HS, Sun C, Yang SJ, et al. Randomized, Open-Label, Parallel Group Study to Evaluate the Effect of Internet-Based Glucose Management System on Subjects with Diabetes in China. Telemed J E Health. 2016;22(8):666-674. doi:10.1089/tmj.2015.0170 |
| 14 | Gerber BS, Biggers A, Tilton JJ, et al. Mobile Health Intervention in Patients With Type 2 Diabetes: A Randomized Clinical Trial. JAMA Netw Open. 2023;6(9):e2333629. Published 2023 Sep 5. doi:10.1001/jamanetworkopen.2023.33629 |
| 15 | Cohen LB, Taveira TH, Wu WC, Pirraglia PA. Pharmacist-led telehealth disease management program for patients with diabetes and depression. J Telemed Telecare. 2020;26(5):294-302. doi:10.1177/1357633X18822575 |
| 16 | Heald AH, Roberts S, Albeda Gimeno L, et al. A Randomised Control Trial to Explore the Impact and Efficacy of the Healum Collaborative Care Planning Software and App on Condition Management in the Type 2 Diabetes Mellitus Population in NHS Primary Care. Diabetes Ther. 2023;14(6):977-988. doi:10.1007/s13300-023-01404-6 |
| 17 | Jeong JY, Jeon JH, Bae KH, et al. Smart Care Based on Telemonitoring and Telemedicine for Type 2 Diabetes Care: Multi-Center Randomized Controlled Trial. Telemed J E Health. 2018;24(8):604-613. doi:10.1089/tmj.2017.0203 |
| 18 | Wild SH, Hanley J, Lewis SC, et al. Supported Telemonitoring and Glycemic Control in People with Type 2 Diabetes: The Telescot Diabetes Pragmatic Multicenter Randomized Controlled Trial [published correction appears in PLoS Med. 2016 Oct 19;13(10):e1002163. doi: 10.1371/journal.pmed.1002163]. PLoS Med. 2016;13(7):e1002098. Published 2016 Jul 26. doi:10.1371/journal.pmed.1002098 |
| 19 | Crowley MJ, Bosworth HB, Coffman CJ, et al. Tailored Case Management for Diabetes and Hypertension (TEACH-DM) in a community population: study design and baseline sample characteristics. Contemp Clin Trials. 2013;36(1):298-306. doi:10.1016/j.cct.2013.07.010 |
| 20 | Castelnuovo G, Manzoni GM, Cuzziol P, et al. TECNOB: study design of a randomized controlled trial of a multidisciplinary telecare intervention for obese patients with type-2 diabetes. BMC Public Health. 2010;10:204. Published 2010 Apr 23. doi:10.1186/1471-2458-10-204 |
| 21 | Lehmkuhl HD, Storch EA, Cammarata C, et al. Telehealth behavior therapy for the management of type 1 diabetes in adolescents. J Diabetes Sci Technol. 2010;4(1):199-208. Published 2010 Jan 1. doi:10.1177/193229681000400125 |
| 22 | Calikoglu F, Bagdemir E, Celik S, et al. Telemedicine as a Motivational Tool to Optimize Metabolic Control in Patients with Diabetes in Turkey: A Prospective, Randomized, Controlled TeleDiab Trial. Telemed J E Health. 2023;29(4):518-530. doi:10.1089/tmj.2022.0028 |
| 23 | Lee JY, Chan CKY, Chua SS, et al. Telemonitoring and Team-Based Management of Glycemic Control on People with Type 2 Diabetes: a Cluster-Randomized Controlled Trial. J Gen Intern Med. 2020;35(1):87-94. doi:10.1007/s11606-019-05316-9 |
| 24 | Ibrahim N, Treluyer JM, Briand N, Godot C, Polak M, Beltrand J. Text message reminders for adolescents with poorly controlled type 1 diabetes: A randomized controlled trial. PLoS One. 2021;16(3):e0248549. Published 2021 Mar 15. doi:10.1371/journal.pone.0248549 |
| 25 | Rasekaba TM, Furler J, Young D, et al. Using technology to support care in gestational diabetes mellitus: Quantitative outcomes of an exploratory randomised control trial of adjunct telemedicine for gestational diabetes mellitus (TeleGDM). Diabetes Res Clin Pract. 2018;142:276-285. doi:10.1016/j.diabres.2018.05.049 |
| 26 | Arora S, Peters AL, Burner E, Lam CN, Menchine M. Trial to examine text message-based mHealth in emergency department patients with diabetes (TExT-MED): a randomized controlled trial [published correction appears in Ann Emerg Med. 2017 Jun;69(6):802. doi: 10.1016/j.annemergmed.2017.04.041]. Ann Emerg Med. 2014;63(6):745-54.e6. doi:10.1016/j.annemergmed.2013.10.012 |
| 27 | Xia SF, Maitiniyazi G, Chen Y, et al. Web-Based TangPlan and WeChat Combination to Support Self-management for Patients With Type 2 Diabetes: Randomized Controlled Trial. JMIR Mhealth Uhealth. 2022;10(3):e30571. Published 2022 Mar 30. doi:10.2196/30571 |
| 28 | Trief PM, Izquierdo R, Eimicke JP, et al. Adherence to diabetes self care for white, African-American and Hispanic American telemedicine participants: 5 year results from the IDEATel project. Ethn Health. 2013;18(1):83-96. doi:10.1080/13557858.2012.700915 |
| 29 | Roddy MK, Nelson LA, Greevy RA, Mayberry LS. Changes in family involvement occasioned by FAMS mobile health intervention mediate changes in glycemic control over 12 months. J Behav Med. 2022;45(1):28-37. doi:10.1007/s10865-021-00250-w |
| 30 | Jia W, Zhang P, Zhu D, et al. Evaluation of an mHealth-enabled hierarchical diabetes management intervention in primary care in China (ROADMAP): A cluster randomized trial. PLoS Med. 2021;18(9):e1003754. Published 2021 Sep 21. doi:10.1371/journal.pmed.1003754 |
| 31 | Seyed Ahmadi S, Westman K, Pivodic A, et al. The Association Between HbA1c and Time in Hypoglycemia During CGM and Self-Monitoring of Blood Glucose in People With Type 1 Diabetes and Multiple Daily Insulin Injections: A Randomized Clinical Trial (GOLD-4). Diabetes Care. 2020;43(9):2017-2024. doi:10.2337/dc19-2606 |
| 32 | Benson GA, Sidebottom A, Hayes J, et al. Impact of ENHANCED (diEtitiaNs Helping pAtieNts CarE for Diabetes) Telemedicine Randomized Controlled Trial on Diabetes Optimal Care Outcomes in Patients with Type 2 Diabetes. J Acad Nutr Diet. 2019;119(4):585-598. doi:10.1016/j.jand.2018.11.013 |
| 33 | Callan JA, Sereika SM, Cui R, et al. Cognitive Behavioral Therapy (CBT) Telehealth Augmented With a CBT Smartphone Application to Address Type 2 Diabetes Self-Management: A Randomized Pilot Trial. Sci Diabetes Self Manag Care. 2022;48(6):492-504. doi:10.1177/26350106221133027 |
| 34 | Cavan DA, Ziegler R, Cranston I, et al. Automated bolus advisor control and usability study (ABACUS): does use of an insulin bolus advisor improve glycaemic control in patients failing multiple daily insulin injection (MDI) therapy? [NCT01460446]. BMC Fam Pract. 2012;13:102. Published 2012 Oct 13. doi:10.1186/1471-2296-13-102 |
| 35 | Feig DS, Donovan LE, Corcoy R, et al. Continuous glucose monitoring in pregnant women with type 1 diabetes (CONCEPTT): a multicentre international randomised controlled trial [published correction appears in Lancet. 2017 Nov 25;390(10110):2346. doi: 10.1016/S0140-6736(17)32712-5]. Lancet. 2017;390(10110):2347-2359. doi:10.1016/S0140-6736(17)32400-5 |
| **Control group does not meet the criteria (n=8)** | |
| 1 | Patnaik, L., Panigrahi, S. K., Sahoo, A. K., Mishra, D., Beura, S., & Muduli, A. K. (2021). Mobile health application based intervention for improvement of quality of life among newly diagnosed type 2 diabetes patients. Clinical Diabetology, 10(3), 276-283. |
| 2 | Shalitin S, Ben-Ari T, Yackobovitch-Gavan M, et al. Using the Internet-based upload blood glucose monitoring and therapy management system in patients with type 1 diabetes. Acta Diabetol. 2014;51(2):247-256. doi:10.1007/s00592-013-0510-x |
| 3 | Tildesley HD, Wright AM, Chan JH, et al. A comparison of internet monitoring with continuous glucose monitoring in insulin-requiring type 2 diabetes mellitus. Can J Diabetes. 2013;37(5):305-308. doi:10.1016/j.jcjd.2013.05.006 |
| 4 | Skrøvseth SO, Årsand E, Godtliebsen F, Joakimsen RM. Data-Driven Personalized Feedback to Patients with Type 1 Diabetes: A Randomized Trial. Diabetes Technol Ther. 2015;17(7):482-489. doi:10.1089/dia.2014.0276 |
| 5 | Benhamou PY, Franc S, Reznik Y, et al. Closed-loop insulin delivery in adults with type 1 diabetes in real-life conditions: a 12-week multicentre, open-label randomised controlled crossover trial. Lancet Digit Health. 2019;1(1):e17-e25. doi:10.1016/S2589-7500(19)30003-2 |
| 6 | Burner E, Mercado J, Hernandez-Saenz A, et al. Design and patient characteristics of the randomized controlled trial TExT-MED + FANS A test of mHealth augmented social support added to a patient-focused text-messaging intervention for emergency department patients with poorly controlled diabetes. Contemp Clin Trials. 2019;80:1-8. doi:10.1016/j.cct.2019.03.003 |
| 7 | Garcia-Tirado J, Brown SA, Laichuthai N, et al. Anticipation of Historical Exercise Patterns by a Novel Artificial Pancreas System Reduces Hypoglycemia During and After Moderate-Intensity Physical Activity in People with Type 1 Diabetes. Diabetes Technol Ther. 2021;23(4):277-285. doi:10.1089/dia.2020.0516 |
| 8 | Hannon TS, Yazel-Smith LG, Hatton AS, et al. Advancing diabetes management in adolescents: Comparative effectiveness of mobile self-monitoring blood glucose technology and family-centered goal setting. Pediatr Diabetes. 2018;19(4):776-781. doi:10.1111/pedi.12648 |
| **Insufficient intervention duration or missing data on intervention duration（n= 8）** | |
| 1 | Xu R, Xing M, Javaherian K, Peters R, Ross W, Bernal-Mizrachi C. Improving HbA1c with Glucose Self-Monitoring in Diabetic Patients with EpxDiabetes, a Phone Call and Text Message-Based Telemedicine Platform: A Randomized Controlled Trial. Telemed J E Health. 2020;26(6):784-793. doi:10.1089/tmj.2019.0035 |
| 2 | Kim Y, Lee H, Seo JM. Integrated Diabetes Self-Management Program Using Smartphone Application: A Randomized Controlled Trial. West J Nurs Res. 2022;44(4):383-394. doi:10.1177/0193945921994912 |
| 3 | Holmen H, Torbjørnsen A, Wahl AK, et al. A Mobile Health Intervention for Self-Management and Lifestyle Change for Persons With Type 2 Diabetes, Part 2: One-Year Results From the Norwegian Randomized Controlled Trial RENEWING HEALTH. JMIR Mhealth Uhealth. 2014;2(4):e57. Published 2014 Dec 11. doi:10.2196/mhealth.3882 |
| 4 | Rosal MC, Ockene IS, Restrepo A, et al. Randomized trial of a literacy-sensitive, culturally tailored diabetes self-management intervention for low-income latinos: latinos en control. Diabetes Care. 2011;34(4):838-844. doi:10.2337/dc10-1981 |
| 5 | Nayak A, Vakili S, Nayak K, et al. Use of Voice-Based Conversational Artificial Intelligence for Basal Insulin Prescription Management Among Patients With Type 2 Diabetes: A Randomized Clinical Trial. JAMA Netw Open. 2023;6(12):e2340232. Published 2023 Dec 1. doi:10.1001/jamanetworkopen.2023.40232 |
| 6 | Murray E, Sweeting M, Dack C, et al. Web-based self-management support for people with type 2 diabetes (HeLP-Diabetes): randomised controlled trial in English primary care. BMJ Open. 2017;7(9):e016009. Published 2017 Sep 27. doi:10.1136/bmjopen-2017-016009 |
| 7 | Orsama AL, Lähteenmäki J, Harno K, et al. Active assistance technology reduces glycosylated hemoglobin and weight in individuals with type 2 diabetes: results of a theory-based randomized trial. Diabetes Technol Ther. 2013;15(8):662-669. doi:10.1089/dia.2013.0056 |
| 8 | Bradway M, Pfuhl G, Joakimsen R, Ribu L, Grøttland A, Årsand E. Analysing mHealth usage logs in RCTs: Explaining participants' interactions with type 2 diabetes self-management tools. PLoS One. 2018;13(8):e0203202. Published 2018 Aug 30. doi:10.1371/journal.pone.0203202 |
| **No reported glycated hemoglobin (HbA1c) data or related data missing (n=24 )** | |
| 1 | Yew TW, Chi C, Chan SY, et al. A Randomized Controlled Trial to Evaluate the Effects of a Smartphone Application-Based Lifestyle Coaching Program on Gestational Weight Gain, Glycemic Control, and Maternal and Neonatal Outcomes in Women With Gestational Diabetes Mellitus: The SMART-GDM Study. Diabetes Care. 2021;44(2):456-463. doi:10.2337/dc20-1216 |
| 2 | Di Molfetta S, Patruno P, Cormio S, et al. A telemedicine-based approach with real-time transmission of blood glucose data improves metabolic control in insulin-treated diabetes: the DIAMONDS randomized clinical trial. J Endocrinol Invest. 2022;45(9):1663-1671. doi:10.1007/s40618-022-01802-w |
| 3 | Arora S, Peters AL, Burner E, Lam CN, Menchine M. Trial to examine text message-based mHealth in emergency department patients with diabetes (TExT-MED): a randomized controlled trial [published correction appears in Ann Emerg Med. 2017 Jun;69(6):802. doi: 10.1016/j.annemergmed.2017.04.041]. Ann Emerg Med. 2014;63(6):745-54.e6. doi:10.1016/j.annemergmed.2013.10.012 |
| 4 | Hermanns N, Ehrmann D, Finke-Groene K, et al. Use of smartphone application versus written titration charts for basal insulin titration in adults with type 2 diabetes and suboptimal glycaemic control (My Dose Coach): multicentre, open-label, parallel, randomised controlled trial. Lancet Reg Health Eur. 2023;33:100702. Published 2023 Jul 24. doi:10.1016/j.lanepe.2023.100702 |
| 5 | Park SW, Kim G, Hwang YC, Lee WJ, Park H, Kim JH. Validation of the effectiveness of a digital integrated healthcare platform utilizing an AI-based dietary management solution and a real-time continuous glucose monitoring system for diabetes management: a randomized controlled trial. BMC Med Inform Decis Mak. 2020;20(1):156. Published 2020 Jul 10. doi:10.1186/s12911-020-01179-x |
| 6 | Whitehouse CR, Knowles M, Long JA, et al. Digital Health and Community Health Worker Support for Diabetes Management: a Randomized Controlled Trial. J Gen Intern Med. 2023;38(1):131-137. doi:10.1007/s11606-022-07639-6 |
| 7 | Yang Y, Lee EY, Kim HS, Lee SH, Yoon KH, Cho JH. Effect of a Mobile Phone-Based Glucose-Monitoring and Feedback System for Type 2 Diabetes Management in Multiple Primary Care Clinic Settings: Cluster Randomized Controlled Trial. JMIR Mhealth Uhealth. 2020;8(2):e16266. Published 2020 Feb 26. doi:10.2196/16266 |
| 8 | Yingyaun, K., Charoensri, S., & Pongchaiyakul, C. (2022). Effect of using Mobile Messenger for Insulin Injection Education for Glycemic Control: A Randomized Controlled Trial. Pacific Rim International Journal of Nursing Research, 26(3). |
| 9 | Munda A, Mlinaric Z, Jakin PA, Lunder M, Pongrac Barlovic D. Effectiveness of a comprehensive telemedicine intervention replacing standard care in gestational diabetes: a randomized controlled trial. Acta Diabetol. 2023;60(8):1037-1044. doi:10.1007/s00592-023-02099-8 |
| 10 | Secher AL, Pedersen-Bjergaard U, Svendsen OL, et al. Flash glucose monitoring and automated bolus calculation in type 1 diabetes treated with multiple daily insulin injections: a 26 week randomised, controlled, multicentre trial [published correction appears in Diabetologia. 2022 Jan;65(1):256. doi: 10.1007/s00125-021-05601-5]. Diabetologia. 2021;64(12):2713-2724. doi:10.1007/s00125-021-05555-8 |
| 11 | McLeod M, Stanley J, Signal V, et al. Impact of a comprehensive digital health programme on HbA1c and weight after 12 months for people with diabetes and prediabetes: a randomised controlled trial. Diabetologia. 2020;63(12):2559-2570. doi:10.1007/s00125-020-05261-x |
| 12 | Miremberg H, Ben-Ari T, Betzer T, et al. The impact of a daily smartphone-based feedback system among women with gestational diabetes on compliance, glycemic control, satisfaction, and pregnancy outcome: a randomized controlled trial. Am J Obstet Gynecol. 2018;218(4):453.e1-453.e7. doi:10.1016/j.ajog.2018.01.044 |
| 13 | Turnin MC, Gourdy P, Martini J, et al. Impact of a Remote Monitoring Programme Including Lifestyle Education Software in Type 2 Diabetes: Results of the Educ@dom Randomised Multicentre Study. Diabetes Ther. 2021;12(7):2059-2075. doi:10.1007/s13300-021-01095-x |
| 14 | Huang F, Wu X, Xie Y, et al. An automated structured education intervention based on a smartphone app in Chinese patients with type 1 diabetes: a protocol for a single-blinded randomized controlled trial. Trials. 2020;21(1):944. Published 2020 Nov 23. doi:10.1186/s13063-020-04835-9 |
| 15 | Ku EJ, Park JI, Jeon HJ, Oh T, Choi HJ. Clinical efficacy and plausibility of a smartphone-based integrated online real-time diabetes care system via glucose and diet data management: a pilot study. Intern Med J. 2020;50(12):1524-1532. doi:10.1111/imj.14738 |
| 16 | Huo X, Spatz ES, Ding Q, et al. Design and rationale of the Cardiovascular Health and Text Messaging (CHAT) Study and the CHAT-Diabetes Mellitus (CHAT-DM) Study: two randomised controlled trials of text messaging to improve secondary prevention for coronary heart disease and diabetes [published correction appears in BMJ Open. 2018 Jan 21;8(1):e018302corr1. doi: 10.1136/bmjopen-2017-018302corr1]. BMJ Open. 2017;7(12):e018302. Published 2017 Dec 21. doi:10.1136/bmjopen-2017-018302 |
| 17 | Kempf K, Dubois C, Arnold M, et al. Effectiveness of the Telemedical Lifestyle Intervention Program TeLIPro for Improvement of HbA1c in Type 2 Diabetes: A Randomized-Controlled Trial in a Real-Life Setting. Nutrients. 2023;15(18):3954. Published 2023 Sep 12. doi:10.3390/nu15183954 |
| 18 | Bonn SE, Alexandrou C, Hjörleifsdottir Steiner K, et al. App-technology to increase physical activity among patients with diabetes type 2 - the DiaCert-study, a randomized controlled trial. BMC Public Health. 2018;18(1):119. Published 2018 Jan 10. doi:10.1186/s12889-018-5026-4 |
| 19 | Dwibedi C, Abrahamsson B, Rosengren AH. Effect of Digital Lifestyle Management on Metabolic Control and Quality of Life in Patients with Well-Controlled Type 2 Diabetes. Diabetes Ther. 2022;13(3):423-439. doi:10.1007/s13300-022-01214-2 |
| 20 | Dwibedi C, Mellergård E, Gyllensten AC, et al. Effect of self-managed lifestyle treatment on glycemic control in patients with type 2 diabetes. NPJ Digit Med. 2022;5(1):60. Published 2022 May 11. doi:10.1038/s41746-022-00606-9 |
| 21 | Han CY, Lim SL, Ong KW, Johal J, Gulyani A. Behavioral Lifestyle Intervention Program Using Mobile Application Improves Diet Quality in Adults With Prediabetes (D'LITE Study): A Randomized Controlled Trial. J Acad Nutr Diet. 2024;124(3):358-371. doi:10.1016/j.jand.2023.10.005 |
| 22 | Harte R, Norton L, Whitehouse C, et al. Design of a randomized controlled trial of digital health and community health worker support for diabetes management among low-income patients. Contemp Clin Trials Commun. 2021;25:100878. Published 2021 Dec 9. doi:10.1016/j.conctc.2021.100878 |
| 23 | Homko CJ, Deeb LC, Rohrbacher K, et al. Impact of a telemedicine system with automated reminders on outcomes in women with gestational diabetes mellitus. Diabetes Technol Ther. 2012;14(7):624-629. doi:10.1089/dia.2012.0010 |
| 24 | Agarwal P, Mukerji G, Desveaux L, et al. Mobile App for Improved Self-Management of Type 2 Diabetes: Multicenter Pragmatic Randomized Controlled Trial. JMIR Mhealth Uhealth. 2019;7(1):e10321. Published 2019 Jan 10. doi:10.2196/10321 |
| **Not relevant to the topic or not a randomized controlled trial (n=2)** | |
| 1 | Xue Y, Zhu Y, Shen J, et al. The Association of Thyroid Hormones With Cardiogenic Shock and Prognosis in Patients with ST Segment Elevation Myocardial Infarction (STEMI) Treated with Primary PCI. Am J Med Sci. 2022;363(3):251-258. doi:10.1016/j.amjms.2021.06.020 |
| 2 | Shen Y, Wang F, Zhang X, et al. Effectiveness of Internet-Based Interventions on Glycemic Control in Patients With Type 2 Diabetes: Meta-Analysis of Randomized Controlled Trials. J Med Internet Res. 2018;20(5):e172. Published 2018 May 7. doi:10.2196/jmir.9133 |

# PRISMA checklist

| **Section and Topic** | **Item #** | **Checklist item** | **Location where item is reported** |
| --- | --- | --- | --- |
| **TITLE** | | |  |
| Title | 1 | Identify the report as a systematic review. | Title; page 1 |
| **ABSTRACT** | | |  |
| Abstract | 2 | See the PRISMA 2020 for Abstracts checklist. | Abstract; page  1-2 |
| **INTRODUCTION** | | |  |
| Rationale | 3 | Describe the rationale for the review in the context of existing knowledge. | Introduction;  page 3-4 |
| Objectives | 4 | Provide an explicit statement of the objective(s) or question(s) the review addresses. | Introduction;  page 4 |
| **METHODS** | | |  |
| Eligibility criteria | 5 | Specify the inclusion and exclusion criteria for the review and how studies were grouped for the syntheses. | Methods; page  4-5 |
| Information sources | 6 | Specify all databases, registers, websites, organisations, reference lists and other sources searched or consulted to identify studies. Specify the date when each source was last searched or consulted. | Methods; page  4 |
| Search strategy | 7 | Present the full search strategies for all databases, registers and websites, including any filters and limits used. | Supplementary  Information S1 Table |
| Selection process | 8 | Specify the methods used to decide whether a study met the inclusion criteria of the review, including how many reviewers screened each record and each report retrieved, whether they worked independently, and if applicable, details of automation tools used in the process. | Methods; page  4-5 |
| Data collection process | 9 | Specify the methods used to collect data from reports, including how many reviewers collected data from each report, whether they worked independently, any processes for obtaining or confirming data from study investigators, and if applicable, details of automation tools used in the process. | Methods and  Fig 1 |
| Data items | 10a | List and define all outcomes for which data were sought. Specify whether all results that were compatible with each outcome domain in each study were sought (e.g. for all measures, time points, analyses), and if not, the methods used to decide which results to collect. | Methods; page  6 |
|  | 10b | List and define all other variables for which data were sought (e.g. participant and intervention characteristics, funding sources). Describe any assumptions made about any missing or unclear information. | Methods; page  6-7 |
| Study risk of bias assessment | 11 | Specify the methods used to assess risk of bias in the included studies, including details of the tool(s) used, how many reviewers assessed each study and whether they worked independently, and if applicable, details of automation tools used in the process. | Methods; page  7 |
| Effect measures | 12 | Specify for each outcome the effect measure(s) (e.g. risk ratio, mean difference) used in the synthesis or presentation of results. | Methods; page  6 |
| Synthesis methods | 13a | Describe the processes used to decide which studies were eligible for each synthesis (e.g. tabulating the study intervention characteristics and comparing against the planned groups for each synthesis (item #5)). | Methods; page  6-7 |
|  | 13b | Describe any methods required to prepare the data for presentation or synthesis, such as handling of missing summary statistics, or data conversions. | Methods; page  6-7 |
|  | 13c | Describe any methods used to tabulate or visually display results of individual studies and syntheses. | Methods; page  6-7 |
|  | 13d | Describe any methods used to synthesize results and provide a rationale for the choice(s). If meta-analysis was performed, describe the model(s), method(s) to identify the presence and extent of statistical heterogeneity, and software package(s) used. | Methods; page  6-7 |
|  | 13e | Describe any methods used to explore possible causes of heterogeneity among study results (e.g. subgroup analysis, meta-regression). | Methods; page  6-7 |
|  | 13f | Describe any sensitivity analyses conducted to assess robustness of the synthesized results. | Methods; page  6-7 |
| Reporting bias assessment | 14 | Describe any methods used to assess risk of bias due to missing results in a synthesis (arising from reporting biases). | Methods; page  6 |
| Certainty assessment | 15 | Describe any methods used to assess certainty (or confidence) in the body of evidence for an outcome. | Methods; page  6 |
| **RESULTS** | | |  |
| Study selection | 16a | Describe the results of the search and selection process, from the number of records identified in the search to the number of studies included in the review, ideally using a flow diagram. | Results; page  7-8 and Fig 1 |
|  | 16b | Cite studies that might appear to meet the inclusion criteria, but which were excluded, and explain why they were excluded. | Supplementary  Information S2 Table |
| Study characteristics | 17 | Cite each included study and present its characteristics. | Table1,2 |
| Risk of bias in studies | 18 | Present assessments of risk of bias for each included study. | Results; page  18-19 and Fig 4-5 |
| Results of individual studies | 19 | For all outcomes, present, for each study: (a) summary statistics for each group (where appropriate) and (b) an effect estimate and its precision (e.g. confidence/credible interval), ideally using structured tables or plots. | Results; page  15 and Fig 2 |
| Results of syntheses | 20a | For each synthesis, briefly summarise the characteristics and risk of bias among contributing studies. | Results; page  16-18 |
|  | 20b | Present results of all statistical syntheses conducted. If meta-analysis was done, present for each the summary estimate and its precision (e.g. confidence/credible interval) and measures of statistical heterogeneity. If comparing groups, describe the direction of the effect. | Results; page  15-17 |
|  | 20c | Present results of all investigations of possible causes of heterogeneity among study results. | Results; page  15-17 |
|  | 20d | Present results of all sensitivity analyses conducted to assess the robustness of the synthesized results. | Results; page  15-17 and Fig 2-3 |
| Reporting biases | 21 | Present assessments of risk of bias due to missing results (arising from reporting biases) for each synthesis assessed. | Results; page  19 and Fig5 |
| Certainty of evidence | 22 | Present assessments of certainty (or confidence) in the body of evidence for each outcome assessed. | Results; page  15-17 and Fig 2-3 |
| **DISCUSSION** | | |  |
| Discussion | 23a | Provide a general interpretation of the results in the context of other evidence. | Discussion;  page 19-25 |
|  | 23b | Discuss any limitations of the evidence included in the review. | Discussion;  page 19-25 |
|  | 23c | Discuss any limitations of the review processes used. | Discussion;  page 24 |
|  | 23d | Discuss implications of the results for practice, policy, and future research. | Discussion;  page 19-25 |
| **OTHER INFORMATION** | | |  |
| Registration and protocol | 24a | Provide registration information for the review, including register name and registration number, or state that the review was not registered. | Methods; page  4 |
|  | 24b | Indicate where the review protocol can be accessed, or state that a protocol was not prepared. | Methods; page  4 |
|  | 24c | Describe and explain any amendments to information provided at registration or in the protocol. | Methods; page  4-5 |
| Support | 25 | Describe sources of financial or non-financial support for the review, and the role of the funders or sponsors in the review. | Provided during submission, not included in the manuscript. |
| Competing interests | 26 | Declare any competing interests of review authors. | Provided during submission, not included in the manuscript. |
| Availability of data, code and other materials | 27 | Report which of the following are publicly available and where they can be found: template data collection forms; data extracted from included studies; data used for all analyses; analytic code; any other materials used in the review. | Supplementary  Information |

*From:*  Page MJ, McKenzie JE, Bossuyt PM, Boutron I, Hoffmann TC, Mulrow CD, et al. The PRISMA 2020 statement: an updated guideline for reporting systematic reviews. BMJ 2021;372:n71. doi: 10.1136/bmj.n71. This work is licensed under CC BY 4.0. To view a copy of this license, visit <https://creativecommons.org/licenses/by/4.0/>
